# European Stroke Organisation (ESO) guidelines on blood pressure management in acute ischaemic stroke and intracerebral haemorrhage

**DOI:** 10.1177/23969873211012133

**Published:** 2021-05-11

**Authors:** Else Charlotte Sandset, Craig S Anderson, Philip M Bath, Hanne Christensen, Urs Fischer, Dariusz Gąsecki, Avtar Lal, Lisa S Manning, Simona Sacco, Thorsten Steiner, Georgios Tsivgoulis

**Affiliations:** 1 Stroke Unit, Department of Neurology, Oslo University Hospital, Oslo, Norway; 2 The Norwegian Air Ambulance Foundation, Oslo, Norway; 3 The George Institute for Global Health, Faculty of Medicine, University of New South Wales, Sydney, NSW, Australia; 4 The George Institute China at Peking University Health Science Center, Beijing, PR China; 5 Stroke Trials Unit, Division of Clinical Neuroscience, University of Nottingham, Nottingham NG7 2UH, United Kingdom; 6 Department of Neurology, Bispebjerg Hospital & University of Copenhagen, Copenhagen, Denmark; 7 Department of Neurology, Inselspital, Bern University Hospital, University of Bern, Bern, Switzerland; 8 Department of Adult Neurology, Medical University of Gdańsk, Gdańsk, Poland; 9 Methodologist, European Stroke Organisation, Basel, Switzerland; 10 Department of Stroke Medicine, University Hospitals of Leicester NHS Trust, Leicester, United Kingdom; 11 Department of Biotechnological and Applied Clinical Sciences, University of L'Aquila, Italy; 12 Department of Neurology, Frankfurt Hoechst Hospital, Frankfurt, Germany; 13 Department of Neurology, Heidelberg University Hospital, Heidelberg, Germany; 14 Second Department of Neurology, Attikon University Hospital, School of Medicine, National and Kapodistrian University of Athens, Athens, Greece; 15 Department of Neurology, University of Tennessee Health Science Center, Memphis, Tennessee, USA

**Keywords:** ischaemic stroke, intracerebral haemorrhage, blood pressure, hypertension, guidelines, recommendations, antihypertensive, blood pressure lowering

## Abstract

The optimal blood pressure (BP) management in acute ischaemic stroke (AIS) and acute intracerebral haemorrhage (ICH) remains controversial. These European Stroke Organisation (ESO) guidelines provide evidence-based recommendations to assist physicians in their clinical decisions regarding BP management in acute stroke.

The guidelines were developed according to the ESO standard operating procedure and Grading of Recommendations, Assessment, Development, and Evaluation (GRADE) methodology. The working group identified relevant clinical questions, performed systematic reviews and meta-analyses of the literature, assessed the quality of the available evidence, and made specific recommendations. Expert consensus statements were provided where insufficient evidence was available to provide recommendations based on the GRADE approach. Despite several large randomised-controlled clinical trials, quality of evidence is generally low due to inconsistent results of the effect of blood pressure lowering in AIS. We recommend early and modest blood pressure control (avoiding blood pressure levels >180/105 mm Hg) in AIS patients undergoing reperfusion therapies. There is more high-quality randomised evidence for BP lowering in acute ICH, where intensive blood pressure lowering is recommended rapidly after hospital presentation with the intent to improve recovery by reducing haematoma expansion. These guidelines provide further recommendations on blood pressure thresholds and for specific patient subgroups.

There is ongoing uncertainty regarding the most appropriate blood pressure management in AIS and ICH. Future randomised-controlled clinical trials are needed to inform decision making on thresholds, timing and strategy of blood pressure lowering in different acute stroke patient subgroups.

## Introduction

Elevated blood pressure levels (systolic blood pressure ≥140 mm Hg and/or diastolic blood pressure ≥90 mm Hg) are common in patients with acute ischaemic stroke (AIS) and acute intracerebral haemorrhage (ICH), but the pathophysiology of the hypertensive response is poorly understood.^1^ Despite considerable research effort, the optimal post-stroke blood pressure management in these conditions remains controversial and unresolved. As randomised-controlled clinical trials (RCTs) of this topic are limited and challenging, clinical decisions are often made on the basis of observational studies that are prone to bias, confounding and random error.^2–5^ Theoretical concepts and pathophysiological arguments are used to defend arguments for and against alteration of blood pressure in the setting of acute stroke; to reduce the risk of stroke recurrence, cerebral oedema, reperfusion haemorrhage for AIS patients after reperfusion therapies, and reduce haematoma expansion and cerebral oedema in ICH; to avoid impairment of cerebral perfusion to viable ischaemic tissue in the presence of altered autoregulation.^6,7^ Whilst most attention has been focused on the avoidance of hypertension, drug-induced hypertension has been proposed as a potential therapeutic strategy to increase cerebral perfusion in some AIS patients.^8^

Although numerous observational studies have shown that both extremely low and high blood pressure levels are associated with worse outcomes in AIS and ICH patients,^9–12^ there has been inconsistency in results from multiple RCTs of different antihypertensive strategies for acute stroke subtypes.

Here, we update previous guidelines from the European Stroke Organisation (ESO) on the management of acute blood pressure in AIS^13^ and ICH,^14^ this including new RCTs and individual patient data meta-analyses (IPDM) that have been published since 2008^13^ and 2014^14^ respectively. We review current evidence and provide recommendations for blood pressure management (both blood pressure reduction and augmentation) in patients with acute stroke; including recommendations for blood pressure management in the pre-hospital setting, according to eligibility for reperfusion treatment in AIS and separately for patients with acute ICH. Our goal was to facilitate decision-making in these patient groups where there is considerable ongoing uncertainty over optimal post-stroke blood pressure management.

## Methods

This guideline was initiated by the ESO and prepared according to ESO standard operating procedures,^15^ which are based on the Grading of Recommendations, Assessment, Development and Evaluations (GRADE) system.^16^ The ESO Guidelines Board and Executive Committee reviewed the intellectual and financial disclosures of the module working group (MWG) members (Table 1) and approved the composition of the group, which was co-chaired by the first (ECS) and last (GT) authors.

**Table 1.** Disclosures of the working group members.

Author	Discipline and affiliation	Intellectual and financial disclosures
Else Charlotte Sandset	Neurologist, Stroke Unit, Department of Neurology, Oslo University Hospital, Oslo, Norway Senior Researcher,The Norwegian Air Ambulance, Oslo, Norway	Intellectual disclosures: Trial Manager of the SCAST trial International Advisory Board of the RIGHT2 trial International Advisory Board of the INTERACT4 trial Secretary General of the European Stroke Organisation Financial disclosures: None
Craig S. Anderson	Professor of Neurology The George Institute for Global Health, Faculty of Medicine, University of New South Wales, Sydney, NSW, Australia Executive Director The George Institute China at Peking University Health Science Center, Beijing, PR China	Intellectual disclosures: Principle Investigator for INTERACT, ENCHANTED and TRIDENT studies Financial disclosures: Research grants from Takeda China
Philip M. Bath,	Stroke Physician, Stroke Trials Unit, Division Clinical Neuroscience, University of Nottingham, Nottingham NG7 2UH UK	Intellectual disclosures: CI ENOS, RIGHT, RIGHT-2 Member Trial Steering Committees/Advisory Committees: SCAST, INTERACT-1/2, ENCHANTED Financial disclosures: Member advisory Boards - DiaMedica, Moleac, Phagenesis (none relevant to this topic)
Hanne Christensen	Professor of Neurology and Consultant Neurologist Department of Neurology, Bispebjerg Hospital & University of Copenhagen, Copenhagen, Denmark.	Intellectual disclosures: Member Trial Steering Committee ENOS Financial disclosures: Speaker honoraria: Bayer. Daiichi-Sankyo, BMS og Boerhinger. National Coordinating Investigator: Portola and Bayer
Urs Fischer	Prof. for Acute Neurology and Stroke; Co-Chairman Stroke Centre Bern Deputy-Director Clinical Trial Unit Bern University of Bern Switzerland	Intellectual disclosures: Steering Committee Member of the TRIDENT trial Financial disclosures: Consultant for Medtronic, Stryker and CSL Behring (none relevant for this topic)
Dariusz Gąsecki	Neurologist, Stroke Unit, Department of Adult Neurology, Medical Univeristy of Gdańsk, Gdańsk, Poland	Intellectual disclosures: Trial Participant of the SCAST trial Past-President of the European Society of Hypertension Working Group on Hypertension and the Brain Financial disclosures:none
Avtar Lal	Guidelines Methodologist, European Stroke Organisation, Basel, Switzerland	Intellectual disclosures: None Financial disclosures: None
Lisa S. Manning	Stroke Physician, Department of Stroke Medicine, University Hospitals of Leicester NHS Trust, UK	Intellectual disclosures: None Financial disclosures: None
Simona Sacco	Neurologist Department of Biotechnological and Applied Clinical Sciences andBiotechnology, University of L’Aquila, Italy	Intellectual disclosures: Co-chair of the Guideline Board of the European Stroke Organization Financial disclosures: Personal fees as speaker or advisor: Abbott, Allergan, AstraZeneca, Eli Lilly, Novartis, Teva. Research grants: Allergan, Novartis. Non-financial support: Abbott, Allergan, Bayer, Bristol-Myers Squibb, Daiichi-Sankyo, Eli Lilly, Medtronic, Novartis, Pfizer, Starmed, Teva. Fees for CME/education: Medscape
Thorsten Steiner	Neurologist, Neurointensivist, Department of Neurology, Frankfurt Hoechst Hospital, Frankfurt, Germany Scientific co-worker, lecturer, Department of Neurology, Heidelberg University Hospital, Heidelberg, Germany	Intellectual disclosures: ATACH-2, Financial disclosures: Personal fees: Bayer, Boehringer, BMS-Pfizer, Daiichy Sankyo, Alexion
Georgios Tsivgoulis	Professor of Neurology,‘Attikon’ University Hospital,Second Department of Neurology,School of Medicine, National and KapodistrianUniversity of Athens,Athens, Greece & Department of Neurology, University of Tennessee Health Science Center, Memphis, Tennessee, USA	Intellectual disclosures: - Section Editor: “Stroke” journal - Associate Editor: “Therapeutics advances in Neurological Disorders” journal -Chair of European Stroke Organization Industry Roundtable Financial disclosures: - Participation in advisory meetings & satellite symposia for Boehringer-Ingelheim; Novartis, Sanofi, Biogen, Genesis Pharma, Teva, Merck-Serono, Bayer, Daichii-Sankyo, Allergan, Specifar, Actavis, Shire, Medtronic, CSL Behring, Abbvie, Abbott, Takeda, Biomarin. - Unrestricted research grants from Novartis, Genesis Pharma, Teva, Shire, Merck-Serono, Medtronic, Boehringer-Ingelheim, Allergan, Abbott

The MWG undertook the following steps:

1. Produced a list of topics of clinical interest to Guideline users that were agreed by all MWG members, without reference to blood pressure management for secondary stroke prevention, in patients with transient ischaemic attack (TIA), and in children with acute stroke.
2. Eight Patient Intervention Comparator Outcome (PICO) were developed and agreed upon within the MWG following a teleconference and e-mail correspondence.
3. Produced a list of relevant outcomes for which the MWG used the Delphi method to score their importance (mean score from 10 respondents on a scale of 1 to 9).

The list of outcomes in the AIS subgroup were:

- Mortality (90 days or end of follow-up)    Mean score: 8.8/9
- Functional outcome (90 days or the end of follow-up) Mean score: 8.8/9
- Recurrent ischaemic stroke   Mean score: 7.0/9
- Symptomatic ICH        Mean score: 6.9/9
- Quality of life (90 days)     Mean score: 6.4/9
- Neurological deterioration    Mean score 5.5/9 (48 hours) 
- Acute kidney injury       Mean score 4.0/9

The list of outcomes in ICH subgroup were:

- Mortality (90 days or      Mean score: 8.9/9 end of follow-up)
- Functional outcome (90 days or end of follow-up)  Mean score: 8.9/9
- Haematoma expansion     Mean score: 6.9/9
- Quality of life (90 days)     Mean score: 5.4/9
- Neurological deterioration    Mean score 6.4/9 (48 hours)   
- Incident ischaemic stroke    Mean score: 5.4/9
- Recurrent ICH          Mean score: 5.2/9
- Acute kidney injury      Mean score 4.2/9

Based on voting scores, functional outcome and mortality were allocated highest priority for both AIS and ICH and were the only outcomes considered in the meta-analyses. The outcome of recurrent ischaemic stroke was also of interest in the AIS subgroup, while haematoma expansion was the outcome considered important in the ICH subgroup. Unless specified otherwise, ‘excellent’ and ‘good’ outcome were defined as 3-month modified Rankin Scale (mRS) scores of 0–1 and 0–2, respectively. Unless specified otherwise, ‘any better’ functional outcome corresponded to an ordinal shift analysis of the mRS score at 3 months. For the subgroup of patients with suspected stroke in the prehospital setting we used the outcomes favoured in the AIS subgroup since the aetiology in the majority (≈80%-85%) of these patients is ischaemic. The MWG formulated a list of PICO questions according to the ESO Guideline SOP, which were reviewed and subsequently approved by the ESO Guidelines Board and Executive Committee.

 4.The main recommendations were based on a systematic review of RCTs evaluating different blood pressure management strategies in AIS and ICH patients. The literature search was completed on September 30, 2020. We conducted a systematic review in all PICOs resulting in 35 different sets of analyses. Details regarding the search strategies are provided in the Supplement. We also included relevant literature published to February 27, 2021 in the final manuscript.
 5.For each PICO question, a group consisting of three to four MWG members was formed.
 6. MWG members assigned to each PICO independently screened the titles and abstracts of the publications identified by the electronic search and assessed the full text of potentially relevant RCTs. Where there were no RCT data available for a certain topic, systematic reviews of non-randomised studies or key observational studies were identified and considered.
7.Where appropriate, a random-effects meta-analysis was conducted using Stata software version 11.0 (Statacorp), with results summarised as odds ratios (ORs) and 95% confidence interval (CI). Any heterogeneity across studies was assessed using the I^2^ statistic, and heterogeneity was classified as moderate (I^2^ ≥ 30%), substantial (I^2^ ≥ 50%), or considerable (I^2^ ≥ 75%).^17^ The Cochrane Collaboration risk of bias tool was used for the risk of bias assessment.
 8.The results of data analysis were imported into the GRADEpro Guideline Development Tool (McMaster University, 2015; developed by Evidence Prime, Inc.). For each PICO question and each outcome, the risk of bias was assessed and quality of evidence rated as high, moderate, low or very low based on the type of available evidence (randomised or observational studies) and considerations on inconsistency of results, indirectness of evidence, imprecision of results, and risk of bias.^15,16^ GRADE evidence profiles/summary of findings tables were generated using GRADEPro.
 9.Each PICO group addressed their respective question by providing distinct sections. First, *Analysis of current evidence* summarised current pathophysiological considerations and relative recommendations from other scientific societies related to that specific question, followed by a summary and discussion of the results of the identified RCTs. Where there was no RCT, the paragraph described the results of systematic reviews of non-randomised studies. Second, *Additional information* was added when more details on RCTs referred to in the first section were needed, to summarise results of observational studies, or to provide information on key subgroup analyses of the included RCTs and on ongoing or future RCTs. Third, *Evidence-based Recommendations* were provided, these based on the GRADE methodology. The direction, strength and formulation of the recommendation were determined according to the GRADE evidence profiles and the ESO-SOP.^15,16,18^ These recommendations do not apply to planned or ongoing trials. Finally, according to the first addendum to the ESO SOP, *Expert Consensus Statements* were added whenever the PICO group considered that there was insufficient evidence available to provide *Evidence-based Recommendations* where practical guidance is needed for routine clinical practice. In that case, a pragmatic suggestion was provided, with the results of the votes of all MWG members (apart from AL, a methodologist who contributed in literature search and data analysis) on this proposal. Importantly, the suggestions provided in this paragraph should not be mistaken as evidence-based recommendations but rather as the opinion of the MWG members.
10.The Guideline document was subsequently reviewed several times by all MWG members and revised until a consensus was reached. Finally, the Guideline document was reviewed and approved by external reviewers and members of the ESO Guidelines Board and Executive Committee.

## Results

### In patients with suspected acute stroke, does pre-hospital blood pressure lowering with any vasodepressor drug compared to no drug improve outcome?

### Analysis of current evidence

High blood pressure in patients with suspect stroke in the ambulance is common, and blood pressure may vary according to stroke subtype.^19^ High blood pressure is associated with poor short- and long-term functional outcome both patients with AIS and ICH. Treatment-resistant very high blood pressure (>185/110 mm Hg) is a contraindication to intravenous thrombolysis (IVT),^20^ whereas blood pressure lowering in acute ICH has been associated with improved functional outcome and reduced haematoma expansion.^21,22^ Blood pressure management is a relatively low-cost treatment option feasible in the pre-hospital setting, and may contribute to improved outcomes in patients with suspected stroke.^23^ Current ESO and European Academy of Neurology (EAN) guidelines do not recommend blood pressure lowering in the pre-hospital setting^24^ and the American Heart Association (AHA)/American Stroke Association (ASA) have no specific recommendations for blood pressure management for patients with suspected stroke in this setting.^25^

The Rapid Intervention with Glyceryl Trinitrate in Hypertensive Stroke trial (RIGHT) randomised 41 patients with suspected stroke (Face Arm Speech Test score of 2 or 3) and systolic blood pressure ≥140 mm Hg within 4 hours of symptom onset. The intervention, transdermal glyceryl trinitrate (GTN), was administered in the ambulance by trained paramedics. At 90 days, there was a significant improvement in the mRS with a shift of 1 point in favour of treatment with GTN (p = 0.04), but no significant difference in mortality (GTN 4/25 vs no GTN 6/16, p = 0.15).^26^

The RIGHT-2 trial recruited 1149 patients with suspected stroke (Fast Arm Speech Test scores 2 or 3) and systolic blood pressure ≥120 mm Hg) within 4 hours of symptom onset, according to a similar intervention as the pilot trial. There were no differences in mRS scores (mRS > 2) in patients with a final diagnosis of stroke (GTN 286/434 [68%] vs no GTN 282/418 [69%]; p = 0.55) nor in all included patients (GTN 358/568 [66%] vs no GTN 373/581 [67%]; p = 0.88), nor in mortality.^27^

**Figure 1.**
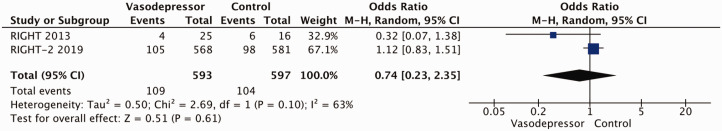
Effect of pre-hospital blood pressure lowering by any vasopressor drug compared to no drug on mortality at three months following symptom onset.

**Figure 2.**
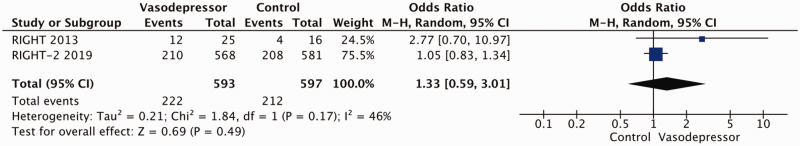
Effect of pre-hospital blood pressure lowering by any vasopressor drug compared to no drug on good functional outcome (mRS scores 0–2) at three months following symptom onset.

Two trials were included in a meta-analysis for the outcome of death at three months,^26^,^27^ with no difference detected between any vasodepressor drug compared to control (OR 0.74, 95%CI: 0.23 – 2.35 p = 0.61, I^2^ = 63%) (Figure 1). There were also no difference in the endpoint of good functional outcome (mRS 0–2 versus 3–6) between any vasodepressor drug compared to control (OR 1.33, 95%CI; 0.59 – 3.01, p = 0.49, I^2^ = 46%) (Figure 2).

Table 2 provides details regarding the safety and efficacy of any vasodepressor drug compared with no vasodepressor drug in patients with suspected stroke in the pre-hospital setting.

**Table 2.** Evidence profile table for pre-hospital blood pressure lowering with any vasodepressor drug compared to no drug in patients with suspected stroke.

Certainty assessment							№ of patients		Effect		Certainty	Importance
№ of studies	Study design	Risk of bias	Inconsistency	Indirectness	Imprecision	Other considerations	PICO 1: Pre-hospital blood pressure lowering	control	Relative (95% CI)	Absolute (95% CI)		
3 months mortality												
2	Randomised trials	Not serious	Not serious	Not serious	Very serious^a^	Publication bias strongly suspected^b^	109/593 (18.4%)	104/597 (17.4%)	OR 0.74 (0.23 to 2.35)	39 fewer per 1,000 (from 128 fewer to 157 more)	⨁◯◯◯ Very low	Critical
3 months good functional outcome (mRS scores 0–2)												
2	Randomised trials	Not serious	Not serious	Not serious	Very serious^a^	Publication bias strongly suspected^b^	222/593 (37.4%)	212/597 (35.5%)	OR 1.33 (0.59 to 3.01)	68 More per 1,000 (from 110 fewer to 269 more)	⨁◯◯◯ Very low	Critical

CI: confidence interval; OR: odds ratio.

^a^Very wide confidence intervals.

^b^Two studies reported this outcome.

### Additional information

The Field Administration of Stroke Therapy-Magnesium (FAST-MAG) randomised 1700 patients in the ambulance with suspected stroke to intravenous magnesium or placebo within 2 hours of symptom onset. The trial evaluated the potential neuroprotective effect of magnesium, which also has mild vasoactive activities. There were no differences between the two groups in the rates of good functional outcome (mRS scores (0-2) at 3 months (magnesium 449/857 [52%) vs placebo 445/843 [53%]; p = 0.88) nor in mortality (magnesium 132/857 [15%] vs placebo 131/843 [16%]; p = 0.95).^28^

The Paramedic Initiated Lisinopril for Acute Stroke Treatment (PIL-FAST) trial randomised 14 patients with unilateral arm weakness, systolic blood pressure ≥160 mm Hg within 3 hours of symptom onset to sublingual lisinopril or control. Blood pressure was lower in the lisinopril group on hospital admission. There was no difference in 1-week mortality between the two groups (lisinopril 1/6 [17%] vs placebo 1/8 [13%]).^23^ Since the trial did not report mortality beyond 1 week, we did not include the trial in the meta-analysis.

In the subgroup of 145 patients included in the RIGHT-2 trial with confirmed ICH after admission to hospital, GTN was associated with a worse shift in the mRS scores (adjusted common OR 1.87, 95%CI 0.98–3.57; p = 0.058). There was no difference in death at days 4 or 90.^29^ Prehospital blood pressure lowering in the ambulance in patients with suspected stroke is currently being tested in two RCTs.^30,31^


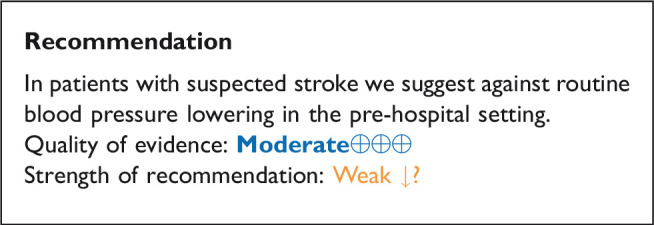

Expert consensus statement

Due to the potential harm in patients with intracerebral haemorrhage prehospital treatment with glyceryl trinitrate should be avoided. Vote 9 of 10.

## In hospitalised patients with acute ischaemic stroke not treated with reperfusion therapies (intravenous thrombolysis or mechanical thrombectomy), does blood pressure lowering with any vasodepressor drug compared to no drug improve outcome?

### Analysis of current evidence

Only a minority of patients with AIS receive reperfusion therapy (intravenous alteplase) in Europe: the percentage ranges from 1% to 20% (mean 7.3%), with even less receiving mechanical thrombectomy (MT) (mean 1.9%).^32^ Based on pathophysiology, optimal blood pressure management may be different in patients who are not eligible for reperfusion treatment with either IVT or MT; lowering blood pressure may reduce the risk of haemorrhagic transformation and oedema, whereas high blood pressure may protect the brain by maintaining cerebral perfusion when autoregulation is partially impaired. Current European^13^ and AHA/ASA^25^ guidelines suggest a cautious approach to hypertension management in those with AIS not eligible for IVT or MT, recommending against aggressive blood pressure lowering in most patients during the initial 24 hours unless blood pressure levels are extreme, or there is a concomitant specific situation that necessitated rapid lowering.

Data from 18 RCTs were considered in the analysis of the effect of blood pressure lowering treatment on death and/or disability.^27,33–50,51,52^ Seven included patients with ICH but reported results separately for ICH and AIS subgroups.^27,37,44,45,47,50,51,52^ One trial did not discriminate between AIS and ICH^34^ but was included based on the assumption that AIS contributed to the majority of patients. Three trials included patients who received IVT, but the proportion receiving IVT was low, and the results were available separately for subgroups according to IVT either in the main paper, or in subsequent post hoc analyses.^27,37,45^ Notably, the majority of studies excluded patients with extremely high systolic blood pressure levels (>220 mm Hg) and many included patients up to 72 hours of symptom onset.

Most of the patients were recruited from the three RCTs described below:

The Scandinavian Candesartan Acute Stroke Trial (SCAST) recruited 2029 patients with AIS (n = 1733) and ICH (n = 274) and systolic blood pressure ≥140 mm Hg within 30 hours of onset and compared treatment with candesartan for seven days with placebo on co-primary end points at six months.^45^ Eight percent of patients with AIS received IVT. Blood pressure was lower in the treatment group at day 7 (−5/−2 mm Hg difference in systolic and diastolic blood pressures). There were neutral effects on the two co-primary endpoints: 1) poor functional outcome at six months (ordinal shift on mRS) and 2) composite outcome of vascular death, myocardial infarction or stroke during the six months follow-up period. There were no significant differences between the candesartan and placebo groups in mortality, functional outcome or recurrent ischaemic stroke in the subgroup of 1733 patient with AIS.^52^

The Efficacy of Nitric Oxide in Stroke trial (ENOS) randomised 4011 patients with AIS (n = 3342), including 225 who received IVT, or ICH (n = 629) and systolic blood pressure 140–220 mm Hg to transdermal GTN patches (5 mg) or placebo within 48 hours of onset for 7 days.^37^ In a partial factorial design, those on pre-existing anti-hypertensive drugs were randomised to stop or continue their medication. Blood pressure was significantly lower in the GTN treatment group at 24 hours (-7/-3 mm Hg difference) but there was no significant difference after day 3. In the overall patient population (AIS and ICH combined) the primary outcome (worse outcome on mRS scores at 90 days, shift analysis) was neutral for GTN versus placebo (adjusted common OR 1.01, 95% CI 0.91 to 1.13, p = 0.83), with no significant interaction between stroke type (AIS versus ICH) and effect of treatment on outcome in a pre-specified subgroup analysis.

The China Antihypertensive Trial in Acute Ischaemic Stroke (CATIS) recruited 4071 patients with AIS (not treated with IVT) and a systolic blood pressure ranging between 140–220 mm Hg within 48 hours of symptom onset.^35^ The study compared targeted blood pressure lowering (10 to 25% systolic blood pressure reduction in 24 hours) with either intravenous angiotensin receptor inhibitors (ACEi) (first line), oral calcium channel antagonists (CCB) (second line), or oral diuretics to control. Mean systolic blood pressure was lower in the treatment group (−9.1 mm Hg at 24 hours). There was no significant effect on functional outcome (mRS ≥3) at 14 days or at 90 days.

All the other included studies reported similarly neutral results.

In analyses of RIGHT-2 limited to 580 patients with AIS, there was no evidence of an effect of GTN on functional outcome at 90 days compared with sham (worse outcome on mRS scores at 90 days, shift analysis); adjusted common OR (1.15 [0.85–1.54]; p = 0.36).^27^

A post hoc subgroup analysis of the Prevention Regime for Effectively Avoiding Second Strokes (PRoFESS) trial examined the effect of adding the angiotensin receptor blocker telmisartan versus placebo to standard antihypertensive treatment in 1360 patients with mild AIS within 72 hours of onset. Telmisartan produced a modest lowering of blood pressure, without increase in adverse events, but there was no effect on poor functional outcome, mortality, stroke recurrence or cardiovascular events. Again, the cohort had mild neurological severity (median National Institutes of Health Stroke Scale (NIHSS) score 3), and treatment initiated rather late (average 58 hours after symptom onset).^43^

In a comparison of amlodipine or irbesartan versus control in 320 AIS patients < 48 hours of onset, the results for effect on poor functional outcome (mRS ≥3) favoured treatment (32.1% in treatment group versus 45.0% in control group, p = 0.018).^42^ Conversely, Intravenous Nimodipine West European Stroke Trial (INWEST), comparing the effect of nimodipine (1 or 2 mg IV for 5 days then 120 mg orally) to placebo in 265 AIS patients reported poorer functional (according to Barthel Index) and neurological outcomes (according to the Orgogozo scales) in the 2 mg treatment group versus placebo at 21 days (primary efficacy time point) and at 24 weeks.^40^

**Figure 3.**
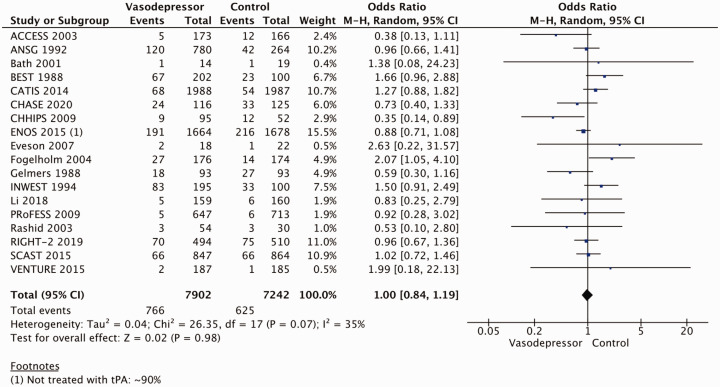
The effect of blood pressure lowering with any vasodepressor drug compared with no drug on mortality at three to six months following symptom onset in patients with acute ischaemic stroke not treated with reperfusion therapies.

**Figure 4.**
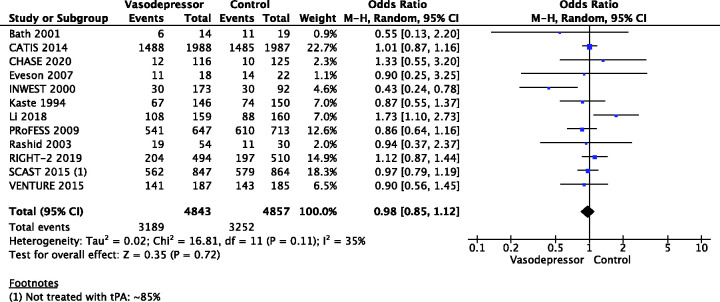
The effect of blood pressure lowering with any vasodepressor drug compared with no drug on good functional outcome (mRS scores 0–2) at three to six months following symptom onset in patients with acute ischaemic stroke not treated with reperfusion therapies.

Eighteen trials were included in the meta-analysis for the effect of blood pressure lowering on mortality at 3–6 months following symptom onset.^27,33--40,42--50,52, 53^ No statistically significant effect was found; OR 1.00 (95%CI: 0.84–1.19), p = 0.98, I^2^ = 35% (Figure 3). Twelve studies were included in the meta-analysis for the effect of blood pressure lowering on improved functional outcome (mRS scores 0 to 2 at three to six months following symptom onset; Figure 4). There was no statistically significant difference between the use of any vasodepressor drug compared with control: (OR 0.98 95%CI: 0.85–1.12, p = 0.72, I^2^ = 35%).^27,35,38,41-47,50,53^

Table 3 provides details regarding the safety and efficacy of any vasodepressor drug compared with no vasodepressor drug in patients with AIS not treated with reperfusion therapies (IVT and/or MT).

**Table 3.** Evidence profile table for blood pressure lowering with any vasodepressor drug compared to no drug in patients with acute ischaemic stroke not treated with reperfusion therapies.

Certainty assessment							№ of patients		Effect		Certainty	Importance
№ of studies	Study design	Risk of bias	Inconsistency	Indirectness	Imprecision	Other considerations	PICO 3 Blood pressure lowering with Vasodepressor	Control	Relative (95% CI)	Absolute (95% CI)		
3-6 months mortality												
18	Randomised trials	Not serious	Not serious	Not serious	Serious^a^	None	766/7902 (9.7%)	625/7242 (8.6%)	OR 1.00 (0.84 to 1.19)	0 Fewer per 1,000 (from 13 fewer to 15 more)	⨁⨁⨁◯ Moderate	Critical
3–6 months good functional outcome (mRS scores 0–2)												
12	Randomised trials	Not serious	Not serious	Not serious	Serious^a^	None	3189/4843 (65.8%)	3252/4857 (67.0%)	OR 0.98 (0.85 to 1.12)	4 Fewer per 1,000 (from 37 fewer to 25 more)	⨁⨁⨁◯ Moderate	Critical

CI: confidence interval; OR: odds ratio.

^a^Wide confidence intervals.

### Additional information

We hypothesise that factors other than blood pressure may influence the treatment effect on outcomes, these including drug class, blood pressure target, timing of treatment, underlying stroke aetiology, premorbid blood pressure levels, and magnitude and rate of blood pressure lowering. Data from the included studies regarding these variables are conflicting. When considering drug class: ACEi appear safe but did not influence outcomes;^35,51^ RCTs using ARBs for blood pressure reduction report conflicting results.^45,46,48^ Candesartan showed promising effects in a pilot trial,^48^ but SCAST was neutral and if anything, favoured placebo;^45^ the Valsartan Efficacy oN modesT blood pressure Reduction in acute ischaemic stroke (VENTURE) trial was neutral for functional outcome but reported significantly more early neurological deterioration among patients in the valsartan group;^46^ the low dose BEta blockade in acute Stroke Trial (BEST) reported increased early death in those randomised to beta-blocker versus control (though this was minimal, and did not reach significance in adjusted analyses),^34^ and in the Controlling Hypertension and Hypotension Immediately Post Stroke (CHHIPS) trial,^51^ labetalol was safe. Results from small RCTs of CCBs have produced mixed results: some favoured placebo,^40,41^ but others have been neutral.^33^ Small and larger RCTs in Nitric Oxide (NO) donors have reported safety with GTN but no significant effect on functional outcome.^29,37,44,47^

Premorbid and initial blood pressure level and blood pressure targets may also be of importance.^1^ Most RCTs excluded AIS patients with extremely elevated systolic blood pressure (>220 mm Hg), and thus the effects of blood pressure lowering in this group are unknown. Owing to heterogeneity in terms of blood pressure targets among studies, no robust conclusions can be drawn as to the optimal blood pressure target, though avoiding large drops in blood pressure during the first 24 hours seems reasonable given the negative effects reported on some outcomes in trials of intravenous CCBs with large (>20%) reduction in blood pressure.^40,41,49,53^ Time to treatment may have an effect and has varied considerably across RCTs (<4 hours to 5 days). In the ENOS patient subgroup treated within 6 hours^54^ treatment with GTN was associated with significantly improved functional outcome, and among a similar group of participants in SCAST there was a non-significant benefit on the composite vascular endpoint in those treated < 6 hours.^55^ In CATIS, subgroup analysis showed a reduction in poor functional outcome in those randomised later (>24 hours) from stroke symptom onset.^35^ In RIGHT-2, where treatment was initiated within 4 hours of onset, GTN was associated with a non-significant trend for a worse functional outcome;^27^ this tendency towards harm was more evident in patients with very early stroke (<1 hour), and severe stroke (Glasgow Coma Scale < 12, NIHSS >12).^27^

Neurological severity and subtype may alter the effect of treatment. In a secondary subgroup analysis of patients with AIS included in SCAST there was a benefit of treatment with candesartan in larger (total and partial anterior circulation), but not smaller (lacunar) AIS,^52^ as shown in another study.^56^ The presence of large vessel occlusion and significant carotid artery stenosis may influence the effect of blood pressure lowering treatment, although data are partly conflicting: a pre-specified subgroup analysis from SCAST of AIS patients with carotid imaging (n = 993) showed that those with severe stenosis (≥70%) treated with candesartan had a trend towards increased risk of stroke progression and poor functional outcome (ordinal shift on the mRS).^57^ Conversely, in ENOS, GTN was shown to be safe across all levels of ipsilateral carotid stenosis among participants with carotid imaging data (n = 2038).^58^

We considered a 2014 Cochrane review relevant to this topic.^6^ In the subgroup of 11,015 patients with AIS there was no benefit of any vasodepressor drug compared with control on the outcome of death and dependency (as reported in the individual trials): OR = 1.00 (95%CI: 0.92–1.08). Furthermore, no differences in treatment effect were observed in subgroups according to drug class, stroke location (cortical versus subcortical), or blood pressure target used. CCBs, ACEi, ARB, beta-blockers and NO donors all lowered blood pressure.^6^


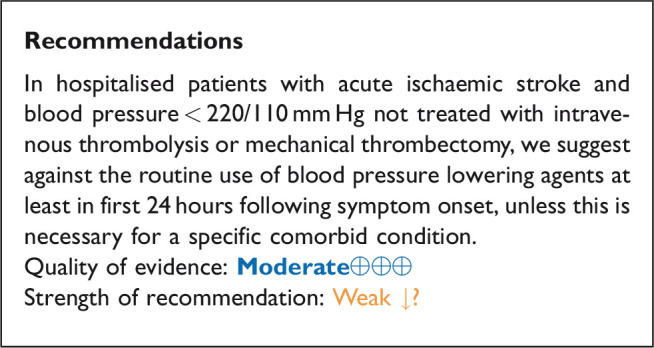

Expert consensus statement

In patients with acute ischaemic stroke not treated with intravenous thrombolysis or mechanical thrombectomy and blood pressure >220/120 mm Hg, careful blood pressure reduction (<15% systolic blood pressure reduction in 24 hours) is reasonable and likely to be safe. No specific blood pressure lowering agent can be recommended. Vote 10 of 10.

## In hospitalised patients with acute ischaemic stroke and undergoing intravenous thrombolysis (with or without mechanical thrombectomy), does blood lowering with any vasodepressor drug compared to control improve outcome?

### Analysis of current evidence

Based on data from small, uncontrolled, non-randomised pilot studies,^59–61^ elevated systolic and diastolic blood pressure levels before (>185/110 mm Hg) and during (>180/105 mm Hg) alteplase infusion of AIS patients are contraindications to IVT based upon the protocol for the original National Institute of Neurological Diseases and Stroke (NINDS)-recombinant tissue plasminogen activator (rt-PA) Stroke Study.^62^ AHA/ASA and ESO guidelines endorse these thresholds and advocate against treating AIS patients with intravenous IVT, when blood pressure is uncontrolled before or during thrombolysis treatment.^13,20,25^

There are no randomised data to support these recommended blood pressure thresholds, and since elevated blood pressure is common in patients with AIS,^63^ IVT can be delayed or even denied in a substantial number of patients whose blood pressure is above this threshold and unresponsive to antihypertensive treatment.^64^ In addition, there is appropriate concern that aggressive blood pressure reduction, may reduce viable penumbral tissue, result in expansion of the cerebral infarction and contribute to neurological deterioration.^64–66^

Conversely, evidence from large, non-randomised real world evidence studies and meta-analyses suggest that elevated blood pressure levels before or during IVT may be related to adverse clinical and imaging outcomes including a higher risk of symptomatic intracranial haemorrhage (sICH), a lower likelihood of complete recanalisation and three-month good functional outcome (mRS scores of 0–2) or three-month functional improvement (1 point decrease across all mRS grades on shift analyses).^67–72^ Additionally, in a post-hoc analysis of the second European Cooperative Acute Stroke Study (ECASS), increasing baseline, maximum and mean (per 10 mm Hg) systolic blood pressure levels were associated with a higher risk of parenchymal haemorrhage during the 7 first days after symptom onset in AIS.^73^ Similarly, in a post-hoc analysis of third International Stroke Trial, the odds of sICH increased by 10% (95%CI: 2–19%) for each 10 mm Hg increase in baseline systolic blood pressure, after adjustment for baseline stroke severity.^74^ Many of these have studies illustrated a linear relationship between increasing systolic or diastolic blood pressure levels and the likelihood of sICH and of death or dependency.^70–74^ They have also indicated that intensive blood pressure control (below the recommended levels of 180/105 mm Hg) during or after alteplase is safe, and may further improve clinical outcomes and reduce the rates of any or symptomatic intracranial bleeding.^67,75,76^

There is no randomised evidence of the safety and efficacy of blood pressure lowering therapies in AIS patients treated with IVT that exceed guideline-recommended blood pressure thresholds (>185/110 mm Hg before alteplase bolus and >180/105 mm Hg during and 24 hours after alteplase infusion).^13,20,25^ However, there is considerable observational data showing that blood pressure protocol violations are common among AIS patients treated with IVT.^67,77,78^ In particular, a single-centre observational study reported that pre-treatment blood pressure violations (>185/110 mm Hg) occurred in 12% of AIS treated with IVT in everyday clinical practice and were independently associated with higher likelihood of sICH (OR: 2.59, 95%CI: 1.07–6.25).^79^ Moreover, in a recent retrospective analysis of the Safe Implementation of Treatments in Stroke (SITS) thrombolysis registry in regard to 11 off-label criteria related to the European license for alteplase, elevated pre-treatment blood pressure levels represented the only off-label criterion that was independently associated with a higher odds of sICH (OR: 1.39; 95%CI: 1.08–1.80).^77^ Finally, a post-hoc analysis of a phase III RCT of sono-thrombolysis that implemented a robust blood pressure control protocol using serial blood pressure recordings before during and after alteplase infusions reported a high rate (34%) of blood pressure excursions above the prespecified thresholds among AIS patients treated with IVT. Most notable was that blood pressure excursions above guideline thresholds were associated with adverse clinical (neurological worsening at 24 hours, functional dependence or death at 3 months) and imaging (any ICH at 24 hours) outcomes.^78^ The heterogeneity of the study populations needs to be considered when interpreting these results: patients had different AIS subtypes (large vessel occlusion vs. lacunar stroke), different medical histories (with or without history of hypertension) and received different blood-pressure lowering therapies (b-blockers vs. calcium channel blockers vs. central acting antihypertensives). Finally, no specific antihypertensive agent has been tested for controlling elevated blood pressure levels (exceeding the recommended thresholds before or during IVT for AIS).

The Enhanced Control of Hypertension and Thrombolysis Stroke Study (ENCHANTED) investigated the safety and efficacy of blood pressure lowering strategies in AIS patients treated with IVT according to guideline criteria (blood pressure levels < 185/110 mm Hg).^80^ 2196 patients with systolic blood pressure > 150 mm Hg who were eligible for IVT with alteplase were randomised to intensive blood pressure lowering (target systolic blood pressure 130–140 mm Hg within 1 hour) or to a standard target systolic blood pressure (<180 mm Hg), and for maintenance of such levels over 72 hours. Mean systolic blood pressure over 24 hours was 144 ± 10 mm Hg in the intensive group and 150 ± 12 mm Hg in the control group.^80^ Functional outcome at 90 days did not differ between groups (unadjusted common OR per 1-point improvement across all mRS scores: 1.01, 95%CI: 0.87–1.17, p = 0.87). However, fewer patients in the intensive group (14.8%) than in the control group (18.7%) had any ICH (OR 0.75, 0.60–0.94, p = 0.01). Blood pressure reduction was also associated with a non-significant decrease in type 2 parenchymal haemorrhage (OR 0.71, 95%CI 0.50–1.01, p = 0.05).^80^ The health-related quality of life was assessed as an overall health utility score (EQ-5D) and no differences were observed between the two treatment groups.^80^

Certain methodological concerns need to be taken into account when interpreting ENCHANTED findings.^81^ First, the study design was open-label and blinded-endpoint adjudication. Second, the mean difference in systolic blood pressure levels during the first 24 hours between the active and control treatment group was modest differing by < 7 mm Hg rather than the planned 15 mm Hg; this may have limited the possibility to detect significant treatment effects between groups. Third, included patients already had blood pressure controlled below the 185/110 mm Hg thresholds and may have received blood pressure lowering treatment prior to inclusion. Fourth, almost three quarters of randomised patients were from Asia where the pattern of cerebrovascular disease differs from the West. Finally, advanced imaging was not included in the selection of patients, and thus the potential of exclusion of patients with higher risk of sICH who may have benefited the most from intensive blood pressure lowering treatment strategies.

Table 4 provides details regarding the safety and efficacy of intensive blood pressure lowering (target systolic blood pressure 130–140 mm Hg within 1 hour) compared to guideline-recommended blood pressure levels (<180 mm Hg) over 72 hours following symptom onset in AIS patients receiving IVT.

**Table 4.** Evidence profile table for safety and efficacy of intensive systolic blood pressure lowering (target 130–140 mmHg within 1 hour) compared to guideline-recommended systolic blood pressure levels (<180 mm Hg) over 72 hours following symptom onset in acute ischaemic stroke patients receiving intravenous thrombolysis.

Certainty assessment							№ of patients		Effect		Certainty	Importance
№ of studies	Study design	Risk of bias	Inconsistency	Indirectness	Imprecision	Other considerations	Experimental arm	Control arm	Relative (95% CI)	Absolute (95% CI)		
3 months mortality												
1	Randomised trial	Unclear	N/A	Not serious	Very serious	N/A	102/1081 (9.4%)	88/1115 (7.9%)	OR 1.22 (0.90 to 1.64)	16 more per 1,000 (from 7 fewer to 44 more)	⨁◯◯◯ Very low	Critical
3 months good functional outcome (mRS scores 0–2)												
1	Randomised trial	Unclear	N/A	Not serious	Very serious	N/A	712/1072 (66.4%)	734/1108 (66.4%)	OR 1.00 (0.83 to 1.20)	0 fewer per 1,000 (from 38 fewer to 42 more)	⨁◯◯◯ Very low	Critical
3 months improved mRS scores (shift analysis)												
1	Randomised trial	Unclear	N/A	Not serious	Very serious	N/A	–	–	common OR 1.01 (0.87 to 1.17)	-	⨁◯◯◯ Very low	Critical

CI: confidence interval; OR: odds ratio.

### Additional information

The ENCHANTED trial observed no significant heterogeneity of the treatment effect (shift on 3-month mRS score) in subgroups including demographics (age, sex, ethnicity), pre-treatment with antiplatelets, dose of alteplase (low vs. standard), stroke severity stratified by NIHSS scores and stroke subgroups where large vessel occlusion might be anticipated, AIS subtypes classified on the basis of clinician diagnosis of large vessel atherosclerosis, cardioembolism or lacunar stroke.^80,82^ Notably, in the prespecified subgroup analysis of severe stroke defined by computed tomography or magnetic resonance angiogram confirmation of large vessel occlusion, receipt of endovascular therapy, final diagnosis of large artery atherosclerotic disease, or high (>10) baseline NIHSS score there was no significant difference in the primary outcome of death or disability at three months in the two treatment arms. However, intensive blood pressure lowering significantly increased three-month mortality (OR 1.52, 95%CI: 1.09–2.13; p = 0.014) compared with guideline blood pressure lowering, despite significantly lower clinician-reported ICH (OR0.63, 95%CI: 0.43–0.92; p = 0.016).^83^ The findings of the aforementioned post-hoc analysis may only serve for hypothesis generation and deserve further validation in future RCTs.

A subgroup analysis of the ENOS trial within 48 hours of stroke onset compared GTN to no GTN in 425 AIS patients presenting with elevated systolic blood pressure levels (140–220 mm Hg) who also received treatment with intravenous alteplase.^37^ A total of 204 and 221 patients were randomised to active and control groups respectively. GTN was not associated with improved three-month functional outcome compared to placebo (unadjusted common OR per 1-point worsening across all mRS scores at three months: 0.93, 95%CI: 0.66–1.30).^37^

An individual patient data meta-analysis of different RCTs evaluating the safety and efficacy of NO for blood pressure management in acute stroke has reported that among 98 AIS patients treated with IVT, GTN was associated with improved functional outcomes at three months compared to placebo (unadjusted common OR per 1-point worsening across all mRS grades: 0.32, 95%CI: 0.15–0.69).^84^ However, this was not confirmed in the RIGHT-2 trial, although the GTN group had a trend towards less haemorrhagic transformation (3% vs 8%; OR: 0.38, 95%CI: 0.13–1.13; p = 0.082).^29^


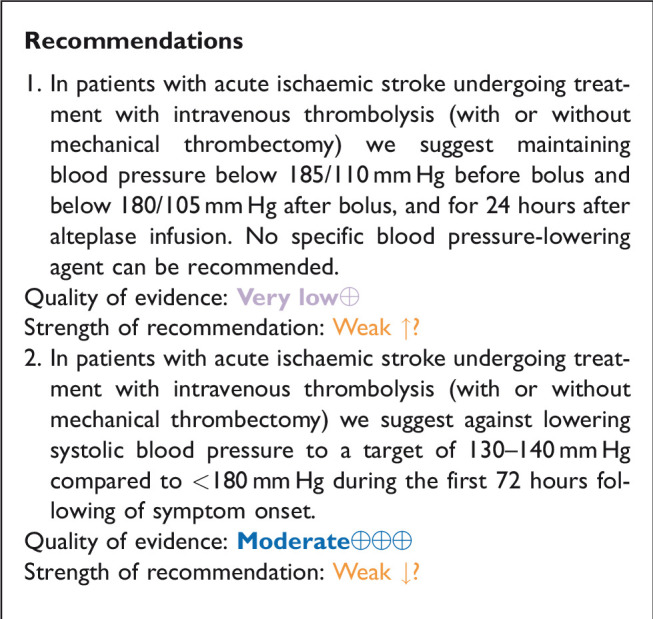


## In patients with acute ischaemic stroke caused by large vessel occlusion and undergoing mechanical thrombectomy (with or without intravenous thrombolysis), does blood pressure lowering with any vasodepressor drug compared to no drug improve outcome?

### Analysis of current evidence

Despite MT becoming standard of care for patients with AIS due to large vessel occlusion of the anterior circulation over the last year,^85–87^ approximately 46% of patients with successful reperfusion die or are left disabled following the procedure.^88^ Blood pressure management in the acute/subacute setting following recanalisation is a potential modifiable determinant of functional improvement in this population. However, data regarding the optimal blood pressure management for AIS undergoing successful MT is scarce^89^ and there is no clear consensus over the intensity of blood pressure (lowering or enhancement) before, during or after the MT.

Current AHA/ASA guidelines advocate thresholds of systolic blood pressure ≤180 mm Hg and diastolic blood pressure ≤105 mm Hg during and for the first 24 hours following MT,^25^ which have been arbitrarily adopted based on evidence regarding blood pressure management in the setting of IVT for AIS. The rationale of this recommendation is to avoid reperfusion haemorrhages associated with elevated blood pressure levels before, during and after MT for large vessel occlusion.

Current MT guidelines from the ESO and European Society for Minimally Invasive Neurological Therapy (ESMINT) recommend systolic and diastolic blood pressure levels < 180/105 mm Hg during and 24 hours after MT and specify the importance of avoiding drops in systolic blood pressure during the procedure.^18^ No specific blood pressure-lowering drug has been recommended. The quality of evidence is graded as low/very low and strength of recommendation is weak in both American^25^ and European^18^ recommendations.

The Society for Neuroscience in Anesthesiology and Critical Care Expert Consensus Statement, recommend that systolic blood pressure should be maintained >140 mm Hg (fluids and vasopressors) and < 180 mm Hg (with or without IVT), and diastolic blood pressure < 105 mm Hg (class IIa, level of evidence B) during endovascular treatment for AIS.^90^

There is a paucity of RCT data regarding the potential safety and efficacy of blood pressure lowering therapies in MT-treated AIS patients with large vessel occlusion that receive endovascular reperfusion therapies. Most large vessel occlusion patients enrolled in the RCTs within 6 hours of symptom onset had received IVT before MT and the trial protocols stipulated management according to local guidelines with systolic or diastolic blood pressure levels ≤180/105 mm Hg during and for 24 hours after the procedure.

#### Observational evidence

Nevertheless, there are observational data indicating that extremely high systolic and/or diastolic blood pressure levels (>220/120 mm Hg) before MT for large vessel occlusion stroke are associated with worse clinical and imaging outcomes including higher rates of sICH, mortality and functional dependence.^91–94^

There are also observational data indicating that hypotension is frequent during MT, can mainly be explained by sedation modality (general anaesthesia in particular) and appears to be associated with infarct expansion and worse functional outcomes at three months.^95^ An analysis of individual patient data from 3 RCTs (SAGA Collaborators) reported that both low (<70 mm Hg) and high (>90 mm Hg) mean arterial blood pressure levels during MT were associated with worse functional outcomes at three months.^96^ Also, a 10% mean arterial blood pressure drop from baseline during MT for AIS has been related to worse 3-month functional outcomes regardless of sedation modality.^97^ Other observational studies and meta-analysis also showed that mean arterial blood pressure falls ≥10% during the endovascular procedure, intra-procedural mean arterial blood pressure levels < 70 mm Hg for a duration of ≥10 min and mean arterial blood pressure < 100 mm Hg before recanalisation are associated with adverse clinical outcomes in MT-treated patients.^98–100^ These observations support the hypothesis that a decrease in systemic blood pressure may lead to hypoperfusion of ischaemic penumbra and cause an increase in final infarction sizes.^99,100^

There are accruing observational evidence that extremely elevated systolic and/or diastolic blood pressure levels after MT for large vessel occlusion stroke appear also to be associated with worse clinical and imaging outcomes including higher rates of sICH, mortality and functional dependence and this association may be mediated by recanalisation status following MT^.^^91,101–104^

Moreover, increased systolic blood pressure variability appears to be associated with adverse clinical outcomes in large vessel occlusion patients treated with MT independent of blood pressure levels.^105^

However, the heterogeneity of study populations needs to be considered when interpreting these results according to recanalisation status (complete versus incomplete or no recanalisation), different blood pressure parameters (systolic/diastolic blood pressure vs. mean arterial blood pressure), medical history (hypertension, cardiac disease), reperfusion strategy (direct thrombectomy vs. bridging therapy with IVT and MT) and different blood pressure lowering agents (β-blockers vs. CCBs vs. centrally acting antihypertensives).

#### Randomised evidence

There is one pilot RCT assessing the feasibility of differential systolic blood pressure targeting during MT for anterior circulation ischaemic stroke. This trial randomly assigned 51 patients to receive either standard or augmented systolic blood pressure management from the start of anaesthesia to recanalisation of the target vessel.^106^ There were no safety concerns with trial procedures and all feasibility targets were achieved.

Further randomised evidence on the intra-procedural management of blood pressure during MT will be provided by an ongoing explorative single-centre RCT with a PROBE (parallel-group, open-label randomised controlled trial with blinded endpoint evaluation) design.^107^ In the control group, intraprocedural systolic blood pressure target range is 140–180 mm Hg. The intervention group is the individualised approach, which is maintaining the intraprocedural systolic blood pressure at the level on presentation (±10 mm Hg). The primary endpoint is the 90-day mRS score dichotomised by 0–2 (good functional outcome) to 3–6 (poor functional outcome). Secondary endpoints include early neurological improvement, infarction size, and systemic physiology monitor parameters. Another ongoing multicentre RCT in France will use PROBE design to assess the individualised approach of blood pressure management during MT.^108^ Patients randomised to the intervention group will be treated with an individualised approach, which is maintaining mean arterial blood pressure during MT within 10% of the first mean arterial blood pressure measurement before MT. In the control group, the intraprocedural systolic blood pressure target range will range between 140–180 mm Hg. The primary endpoint is good functional outcome at 90 days (mRS scores of 0–2). Secondary endpoints include early neurological improvement, excellent outcome, infarct size, systemic physiology monitor parameter, sICH rates and mortality at 3 months.

Table 5 summarises the design of five RCTs that evaluate different blood pressure targets below the pre-specified cut-off of 180/105 mm Hg after the end of MT.^109–112^ The only completed RCT is the Blood Pressure Target in Acute Stroke to Reduce Haemorrhage After Endovascular Therapy (BP-TARGET) trial, a multicentre, prospective, randomised, controlled, open-label, blinded endpoint clinical trial conducted in France.^113,114^ The study enrolled AIS patients with large vessel occlusion in the anterior circulation who had successful reperfusion (defined as modified Thrombolysis In Cerebral Infarction (mTICI) grades of 2 b or 3) following MT. The enrolled patients were randomly assigned, in a 1:1 ratio, to have intensive (systolic blood pressure target 100–129 mm Hg) or a conservative (systolic blood pressure target 130–185 mm Hg) blood pressure control in the following 24 hours, with the primary efficacy endpoint of radiographic intraparenchymal haemorrhage at 24–36 hours and the primary safety endpoint of hypotension occurrence. Secondary endpoints included the rate of the sICH, the overall distribution of the mRS scores at 90 days, good functional outcome (90–day mRS scores of 0–2), functional improvement (90-day decrease by 1 point across all 90-day mRS grades), infarct volume at follow-up CT scan at 24–36 h, change in NIHSS-scores at 24 hours, and all-cause mortality at 90 days. A total of 158 and 160 patients were randomised to the intensive and conservative systolic blood pressure groups respectively, with similar proportions of the primary endpoint (any ICH: 42% in the intensive group vs. 43% in the conservative group) and hypotension in the two treatment groups (8% in the intensive group vs. 3% in the conservative group). All secondary endpoints including three-month functional improvement (common OR for 1-point improvement across all mRS categories: 0.86; 95%CI: 0.57–1.28), three-month good functional outcome (mRS scores 0–2; 44% vs. 45%) and three-month mortality (19% vs. 14%) were similar in the two treatment groups.^113^ The main methodological shortcoming of this RCT included: (i) modest systolic blood pressure difference (10 mm Hg) between the randomised groups; (ii) one third of the individuals in the conservative arm having systolic blood pressure measurements < 130 mm Hg; (iii) moderate sample size; (iv) primary endpoint being imaging, rather than clinical, (v) non-invasive modality of blood pressure assessment every 15 minutes for the first 2 hours, then every 30 minutes for 6 hours and every 1 hour for the remaining 16 hours.

**Table 5.** Randomized controlled clinical trials evaluating different blood pressures targets following mechanical thrombectomy in acute ischaemic stroke patients with large vessel occlusion receiving endovascular therapies.

Study	Location	N patients	Experimental targets	Standard target	Randomization	Period of intervention	Termination date
BEST-II (109)	USA (Cincinnati & Nashville)	120	140–160 mmHg^a^ 110–140 mmHg^b^	160–180 mmHg	N/A	24 hours	March 2023
DETECT(111)	Canada (Hamilton)	30	<140 mmHg	<180 mmHg	1 hour	48 hours	June 2022
ENCHANTED2(110)	International	2236	<120 mmHg	140–180 mmHg	3 hours	72 hours	February, 2023
OPTIMAL BP (112)	Korea (multicenter)	644	<140 mmHg	<180 mmHg	0.5-1 hour	24 hours	December 2023
BP-TARGET (113,114)	France (multicenter)	320	<130 mmHg	<185 mmHg	1 hour	24–36 hours	Completed No differences in clinical or imaging endpoints between the two randomization arms

^a^First active comparator arm of BEST-II.

^b^Second active comparator arm of BEST-II.

Table 6 provides details regarding the safety and efficacy of reducing systolic blood pressure <130 mm Hg in anterior circulation large vessel occlusion during the first 24 hours following successful MT.

**Table 6.** Evidence profile for reducing systolic blood pressure <130mmHg in anterior circulation large vessel occlusion during the first 24 hours following successful mechanical thrombectomy. The following outcomes were evaluated: (i) 3 months mortality; (ii) 3 months good functional outcome (mRS scores 0-2); (iii) 3 months functional improvement (defined as 1-point decrease across all mRS-scores); (iv)any Intracranial Hemorrhage (ICH).

Certainty assessment							№ of patients		Effect		Certainty	Importance
№ of studies	Study design	Risk of bias	Inconsistency	Indirectness	Imprecision	Other considerations	Experimental arm	Control arm	Relative (95% CI)	Absolute (95% CI)		
3 months mortality												
1	Randomised trial	Unclear	N/A	Not serious	Serious	N/A	29/152 (19.1%)	21/153 (13.7%)	OR 1.48 (0.80 to 2.74)	53 More per 1,000 (from 24 fewer to 166 more)	⨁◯◯◯ Very low	Critical
3 months good functional outcome (mRS scores 0–2)												
1	Randomised trial	Unclear	N/A	Not serious	Serious	N/A	67/152 (44.1%)	69/153 (45.1%)	OR 0.96 (0.61 to 1.51)	10 Fewer per 1,000 (from 117 fewer to 103 more)	⨁◯◯◯ Very low-	Critical
3 months improved mRS scores (shift analysis)												
1	Randomised trial	Unclear	N/A	Not serious	Serious	N/A	–	–	Common OR 0.86 (0.57 to 1.28)	–	⨁◯◯◯ Very low	Critical
Any ICH												
1	randomised trial	Unclear	N/A	Not serious	serious	none	65/154 (42.2%)	68/157 (43.3%)	OR 0.96 (0.60 to 1.51)	10 fewer per 1,000 (from 115 fewer to 101 more)	⨁◯◯◯ Very low	Important

CI: confidence interval; OR: odds ratio.

### Additional information

Two protocols of RCTs evaluating the safety and efficacy of MT compared to standard therapy in AIS patients with anterior circulation large vessel occlusion have provided additional recommendations regarding optimal blood pressure management before and after the endovascular procedure. The ESCAPE (Endovascular Treatment for Small Core and Proximal Occlusion Ischaemic Stroke) trial protocol stated that systolic blood pressure ≥150 mm Hg is probably useful in promoting and keeping collateral flow adequate, while the artery remained occluded, and that controlling blood pressure once reperfusion has been achieved and aiming for a normal blood pressure for that individual was sensible.^115^ Labetalol or an intravenous beta-blocker such as metoprolol in low doses were recommended for post-procedural blood pressure management.^115^ The DAWN (Clinical Mismatch in the Triage of Wake Up and Late Presenting Strokes Undergoing Neurointervention With Trevo) trial protocol recommended maintaining systolic blood pressure < 140 mm Hg in the first 24 hours in subjects who were reperfused after MT.^116^

Three RCTs investigating the optimal anaesthesia management (conscious sedation vs. general anaesthesia) of large vessel occlusion patients treated with MT have suggested specific systolic blood pressure ranges during the procedures.^117–119^ The advocated intraprocedural systolic blood pressure range until reperfusion was 140–160 mm Hg in SIESTA (Sedation vs. Intubation for Endovascular Stroke TreAtment) trial,^117^ 140–180 mm Hg in ANSTROKE (Anaesthesia During Stroke) trial^118^ and >140 mm Hg in GOLIATH (General Or Local Anaesthesia in Intra Arterial THerapy) trial.^119^

Large vessel occlusion patients who undergo successful reperfusion documented as mTICI grades ≥2 b following endovascular therapy display spontaneous blood pressure drop due to potential earlier reperfusion of the ischaemic penumbra leading to earlier haemodynamic normalisation.^120,121^ In addition, different observational studies have evaluated blood pressure levels following successful recanalisation in AIS patients due to large vessel occlusion and have consistently reported an inverse association between increasing post-reperfusion blood pressures and good functional outcomes at three months.^122–124^ They also highlight, that systolic blood pressure levels < 140 mm Hg post MT are related to the higher odds of good functional outcome. Also, a single-centre study has recently documented that spontaneous systolic blood pressure drop after MT is an early predictor of dramatic neurological recovery (defined as 8-point-reduction in baseline NIHSS-score or an overall NIHSS ≤ 2 points at 24 h) in large vessel occlusion patients that receive endovascular treatment.^125^ There is no randomised data regarding the safety and efficacy of induced hypertension in large vessel occlusion patients who achieve successful reperfusion following endovascular therapy. Nevertheless, there are theoretical concerns against drug-induced hypertension in large vessel occlusion with complete recanalisation (in particular in patients achieving mTICI 3 corresponding to complete reperfusion without any parenchymal defects even in distal vessels) following endovascular therapy. In particular, transcranial Doppler studies monitoring cerebral haemodynamics of AIS patients in real-time have shown that elevated flow velocities following complete recanalisation of large vessel occlusion may lead to hyperperfusion haemorrhage within the area of infarcted brain tissue in large vessel occlusion patients achieving mTICI 2 b/2c/3 grades following MT.^126–128^

Dynamic autoregulation-based blood pressure targets instead of fixed blood pressure thresholds hold promise as a guide for individualising haemodynamic management in AIS due to large vessel occlusion. In a small observational study, a novel approach to define the limits of autoregulation using near-infrared spectroscopy (NIRS) was proposed. Continuous non-invasive NIRS monitoring in response to changes in mean arterial pressure was found to identify and track the patient-specific blood pressure range at which autoregulation was optimally functioning in individual patients after large vessel AIS.^129^


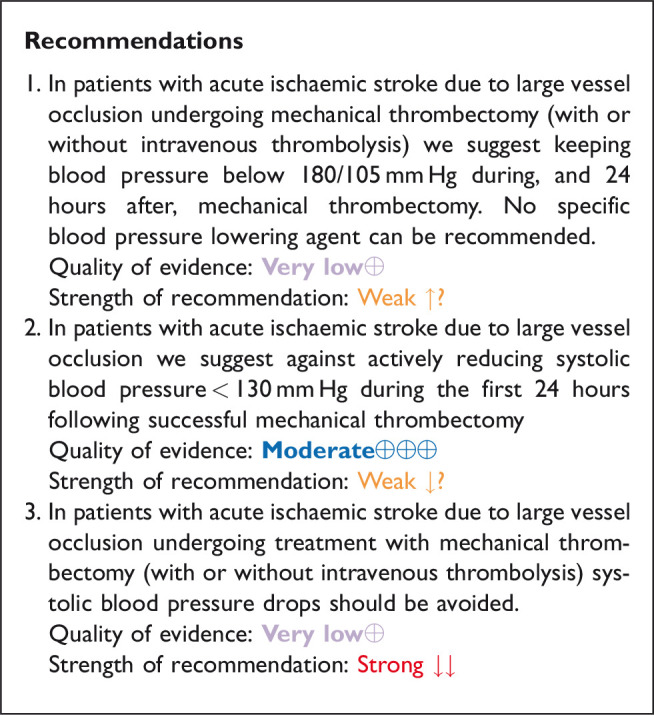

Expert consensus statement

In patients with acute ischaemic stroke due to large vessel occlusion who achieve successful reperfusion defined as modified Thrombolysis in Cerebral Infarction grade of 3 following mechanical thrombectomy we suggest against induced hypertension. Vote 10 of 10

## In patients with acute ischaemic stroke not treated with reperfusion therapies (intravenous thrombolysis or mechanical thrombectomy) and with clinical deterioration, does induced hypertension by any vasopressor drug compared to no drug improve outcome?

### Analysis of current evidence

The hypertensive response seen in patients with AIS has traditionally been viewed as a pathophysiological response to ensure adequate cerebral perfusion in patients with compromised cerebral circulation. The rationale for pharmacologically inducing hypertension is to maintain adequate cerebral perfusion especially in patients with large vessel occlusion, fluctuating symptoms and low blood pressure who are not eligible for reperfusion or where reperfusion has failed.

The Safety and Efficacy of Therapeutic Induced HYPERTENSION (SETIN-HYPERTENSION) randomised 153 patients with major neurological deficit (NIHSS-score 4–18) from non-cardioembolic stroke aetiology, ineligibility for reperfusion therapy or progressive stroke. Progressive stroke was defined as 2-point increase in the NIHSS score, including an increase in the motor score for the affected upper or lower limbs during hospitalisation and the presence of new lesions or infarct growth on DWI performed within 24 hour of aggravation.^130^ Patients with systolic blood pressure >170 mm Hg at baseline were excluded. The intervention group received treatment with intravenous phenylephrine with a target of 20% increase in systolic blood pressure from baseline. Induced hypertension was associated with early neurologic improvement at 7 days (OR:2.49, 95%CI: 1.25–4.96, p = 0.010) though, this did not translate into functional improvement at 90 days (shift analysis in mRS scores): unadjusted common OR 1.27, 95%CI: 0.72–2.22, p = 0.422). Mortality at 90 days did not differ between the intervention group (1.3%) and the control group (0%; p = 0.313). The rates of patients with good functional outcome at 90 days tended to be higher in the intervention group (75.0% vs. 63.2%, p = 0.114). ICH on follow-up MRI was more prevalent in the induced hypertension group (6.6% vs 0%, p = 0.022), however the rates of sICH were similar in the two groups (1.3% vs. 0%, p = 0.313).^130^ There are several limitations to the SETIN-HYPERTENSION trial. First, patients in the induced hypertension group were younger, were more often included due to stroke progression, had higher NIHSS scores and a higher rate of large vessel occlusions. Second, the small sample size and that the trial was conducted in Korea, generalisability is questionable.

Table 7 provides details regarding the safety and efficacy of blood pressure elevation using any vasopressor drug compared to no vasopressor drug in patients with AIS and clinical deterioration not eligible for reperfusion treatment.

**Table 7.** Evidence profile for the safety and efficacy of blood pressure elevation using any vasopressor drug compared to no vasopressor drug in patients acute ischaemic stroke and clinical deterioration not treated with reperfusion therapies.

Certainty assessment							№ of patients		Effect		Certainty	Importance
№ of studies	Study design	Risk of bias	Inconsistency	Indirectness	Imprecision	Other considerations	blood pressure elevation with vasopressor	Control	Relative (95% CI)	Absolute (95% CI)		
3 months mortality												
1	Randomised trial	Not serious	Not serious	Not serious	Very serious^a^	Publication bias strongly suspected^b^	1/76 (1.3%)	0/77 (0.0%)	OR 3.08 (0.12 to 76.79)	0 fewer per 1,000 (from 0 fewer to 0 fewer)	⨁◯◯◯ Very low	Critical
3 months good functional outcome (mRS scores 0–2)												
1	Randomised trial	Not serious	Not serious	Not serious	Serious^c^	Publication bias strongly suspected^b^	57/76 (75.0%)	49/77 (63.6%)	OR 1.71 (0.85 to 3.44)	113 more per 1,000 (from 38 fewer to 221 more)	⨁⨁◯◯ Low	Critical

CI: confidence interval; OR: odds ratio.

^a^Very wide confidence intervals.

^b^One study reported this outcome.

^c^Wide confidence intervals.

### Additional information

In a pilot trial, 15 patients recruited within 7 days of symptom onset, with > 20% diffusion – perfusion mismatch on MRI and quantifiable, stable or worsening of aphasia, hemispatial neglect and/or hemiparesis were randomly assigned to induced hypertension with phenylephrine or control.^131^ There was more improvement in NIHSS scores in the induced hypertension group compared to the control group on day 3 and on day 90.^131^ Since functional outcome was not assessed with mRS at 90 days the study was not included in our meta-analysis.

The Early Manipulation of Arterial Blood Pressure in Acute Ischaemic Stroke (MAPAS) trial randomised 218 patients within 12 hours of acute ischaemic stroke to maintain systolic blood pressure during 24 hours within three ranges; Group 1: SBP target 140–160 mm Hg, Group 2: SBP target 161–180 mm Hg, and Group 3: SBP target 181–200 mm Hg.^132^ Overall, systolic blood pressure was increased in 41% of the patients. Norepinephrine was used to increase blood pressure in 17% of patients in Group 1, 48% of patients in Group 2 and 62% of patients in Group 3. There was no difference between the groups in functional outcome at 90 days. Adverse events (acute coronary syndrome and bradycardia) were limited to group 2 (4%) and Group 3 (7.6%) and were associated with norepinephrine infusion. There was also a significantly higher sICH rate in Group 3.^132^

In addition, observational data from small pilot studies indicate that phenylephrine induced hypertension may be associated with neurological improvement in patients with AIS due to large artery atherosclerotic steno-occlusive disease or small vessel occlusion.^133–137^


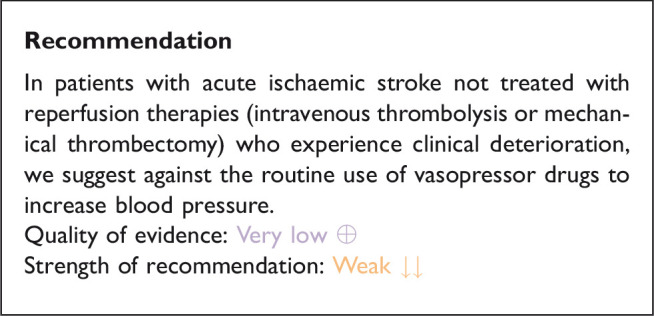

Expert consensus statement

In patients with acute ischaemic stroke not treated with reperfusion therapies (intravenous thrombolysis or mechanical thrombectomy) and with clinical deterioration where a haemodynamic mechanism is suspected or shown to be directly responsible for the deterioration, we suggest:

• stopping existing blood pressure lowering therapy,

• administering intravenous fluids and

• introducing non-pharmacological procedures to raise blood pressure

before considering

• careful use of vasopressor agents to increase blood pressure with close monitoring of blood pressure values. Vote 10 of 10.

## In patients with acute ischaemic stroke, does continuing versus temporarily stopping previous oral blood pressure lowering therapy improve outcome?

### Analysis of current evidence

Blood pressure lowering therapy is a key strategy for the primary and secondary prevention of AIS and other serious cardiovascular events. However, it is unclear if continuing or stopping previous oral antihypertensive agents is beneficial in the AIS setting. Continuing prior antihypertensive agents may improve blood pressure control, however, may also lower blood pressure and worsen the perfusion of the critical hypoperfused brain tissue.

One RCT examined the effect of continuing or stopping previous blood pressure lowering therapy in AIS. The Continue Or Stop post-Stroke Antihypertensives Collaborative Study (COSSACS) was a UK multicentre, prospective, randomised, open, blinded-endpoint trial.^138^ In this study 763 patients who had acute stroke and were taking antihypertensive drugs were enrolled within 48 h of stroke onset. Among the overall group, 444 patients were included with AIS. In the predetermined subgroup analysis of AIS patients in COSSACS there was a benefit in death or dependency at three months (mRS scores ≥3) in the continue group versus the stop group (46 of 241 patients versus 55 of 203 in the stop group; relative risk reduction 0.70, 95% CI 0.51–0.99; p = 0.045).^138^

ENOS was a multicentre, partial-factorial, randomised trial. In this study, 4011 patients who had stroke were enrolled within 48 h of stroke onset. Among the overall group, 928 patients with AIS were randomised to continue and 904 to stop antihypertensive treatment.^37^

**Figure 5.**
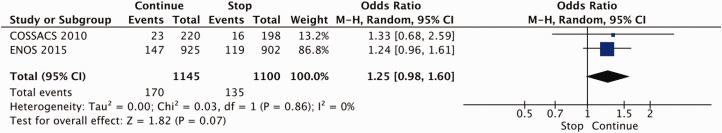
The effect of continuing versus temporarily stopping previous blood pressure lowering therapy on mortality at three to six months following symptom onset in patients with acute ischaemic stroke.

**Figure 6.**
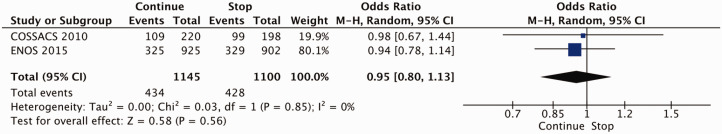
The effect of continuing versus temporarily stopping previous blood pressure lowering therapy on good functional outcome (defined as mRS scores 0–2) at three to six months following symptom onset in patients with acute ischaemic stroke.

A meta-analysis of individual patient data from the COSSACS and Efficacy of Nitric Oxide in Stroke (ENOS) trial evaluated the effect of continuing versus stopping previous blood pressure-lowering therapy on death or dependency and included 2335 patients with AIS. There were no significant associations between continuing versus stopping previous blood pressure lowering therapy and the odds of death or improved functional outcome at 3–6 months in the AIS subgroup.^139^ Two trials^37,138^ were included in the meta-analysis on continuing versus stopping previous blood pressure lowering therapy. There were no significant differences of continuing versus stopping previous blood pressure lowering therapy on mortality (OR 1.25, 95% CI 0.98 – 1.60, p = 0.07, I_2_ = 0%; Figure 5) nor good functional outcome (mRS 0 – 2) (OR 0.95, 95 % CI 0.83 – 1.13, p = 0.56, I2 = 0%; Figure 6).

Table 8 provides details regarding the safety and efficacy of continuing versus temporarily stopping previous blood pressure lowering therapy in AIS patients based on published and unpublished data from the individual patient data meta-analysis.^139^

**Table 8.** Evidence profile table for continuing versus temporarily stopping previous blood-pressure lowering therapy in patients with acute ischemic stroke.

Certainty assessment							№ of patients		Effect		Certainty	Importance
№ of studies	Study design	Risk of bias	Inconsistency	Indirectness	Imprecision	Other considerations	Continuing	Stopping previous antihypertensive therapy	Relative (95% CI)	Absolute (95% CI)		
3–6 months mortality												
2	Randomised trials*	Not serious	Not serious	Not serious	Serious^a^	Publication bias strongly suspected^b^	170/1145 (14.8%)	135/1100 (12.3%)	OR 1.25 (0.98 to 1.60)	26 More per 1,000 (From 2 fewer to 60 more)	⨁⨁◯◯ Low	Critical
3–6 months good functional outcome (mRS scores 0–2)												
2	Randomised trials*	Not serious	Not serious	Not serious	Serious^a^	publication bias strongly suspected^b^	434/1145 (37.9%)	428/1100 (38.9%)	OR 0.95 (0.80 to 1.13)	12 Fewer per 1,000 (From 52 fewer to 29 more)	⨁⨁◯◯ Low	Critical

CI: confidence interval; OR: odds ratio.

^a^Wide confidence intervals.

^b^Two studies reported this outcome

### Additional information

When deciding to continue or stop previous antihypertensive agents whether the patient has received reperfusion treatment, premorbid blood pressure values and the need for antihypertensive agents to maintain blood pressure within the recommended thresholds need to be considered. In case an antihypertensive is needed to maintain blood pressure values within the recommended range, continuation of previous antihypertensive agents may favour more stable blood pressure control. However, the continuation of oral antihypertensive agents may be challenging in patients with dysphagia and impaired consciousness. The route of application depends on the ability to swallow and/or the degree of consciousness.


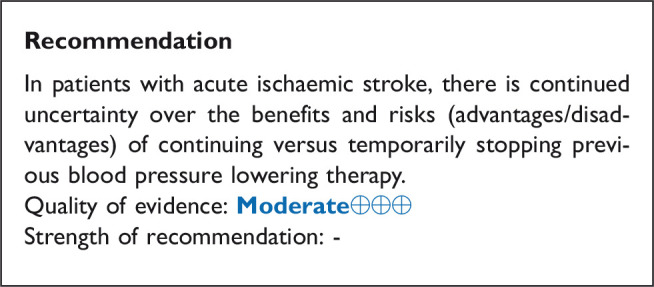

Expert consensus statement

In patients with acute ischaemic stroke we suggest stopping previous oral blood pressure lowering therapy in patients with dysphagia until swallowing is restored or a nasogastric tube is in place. Vote 10 of 10

## In patients with acute intracerebral haemorrhage, does intensive blood pressure lowering with any vasodepressor drug compared to control improve outcome?

### Analysis of current evidence

The rationale behind decreasing blood pressure in acute spontaneous ICH is to reduce the driving force of haematoma expansion and thereby to prevent further clinical deterioration. Most haematoma expansion takes place within the first three hours.^140^

The INTensive blood pressure reduction in Acute Cerebral Haemorrhage trial (INTERACT); compared intensive lowering with a target systolic blood pressure of <140 mm Hg within 1 hour (intensive group) with a moderate lowering with a target systolic blood pressure of <180 mm Hg within 6 hours (standard group) after spontaneous ICH.^141^ The trial recruited 404 patients. Blood pressure lowering drugs were administered according to a “stepped intravenous protocol to lower blood pressure” which was “established before the start of the study based on which drugs were available in that country” with urapidil being the most frequently used agent (47%). The primary efficacy endpoint (the mean proportional change in haematoma volume at 24 hours) was significantly smaller in the intensive lowering group (13.7%) compared to the standard group (36.3%, difference = 22.6%, 95% CI: 0.6–44.5%; p = 0.04).^141^ There was no difference in the risk of adverse events.^141^

INTERACT-2 recruited 2839 patients using the identical treatment protocol as the INTERACT trial. There was no signficant difference in the primary outcome (death or dependency at 90 days defined as mRS scores of 3–6) (OR with intensive treatment, 0.87; 95% CI:0.75–1.01; p = 0.06).^21^ However, the pre-defined secondary endpoint (ordinal analysis of 3-month mRS scores) showed significantly lower mRS scores with intensive treatment (common OR for functional worsening defined as 1-point increase across all mRS scores 0.87; 95% CI: 0.77–1.00; p = 0.04). The rates of non-fatal serious adverse events did not differ between the groups.^21^

The Antihypertensive Treatment of Acute Cerebral Haemorrhage-II **(**ATACH-II) trial compared intensive (target systolic blood pressure 111–140 mm Hg) with moderate (target systolic blood pressure 140–180 mm Hg) blood pressure lowering with intravenous nicardipine in patients with spontaneous ICH within 4.5 hours of symptom onset.^142^ The trial recruited 1000 patients and there were no differences between the intensive and moderate groups in primary endpoint (death or disability at three months defined as mRS-scores of 4–6): adjusted relative risk (RR): 1.04; 95% CI: 0.85–1.27. There was a significantly higher rate of renal adverse events within 7 days after randomisation in the intensive treatment group (9.0% vs. 4.0%, p = 0.002).^142^ A post-hoc-analysis of ATACH-II showed a significant reduction on haematoma expansion within 24 hours and a significantly better functional outcome at three months when blood pressure lowering was initiated within 2 hours of symptom onset.^143^

The Controlling Hypertension After Severe Cerebrovascular Event (CHASE) trial included a mixed population with either severe AIS or ICH patients presenting with systolic blood pressure levels ranging between 150 mm Hg and 210 mm Hg.^50^ The trial compared an individualised blood pressure lowering strategy with standard blood pressure lowering. In the subgroup of ICH (n = 242), the rates of the primary endpoint (the proportion of patients with a poor functional outcome at day 90 of enrolment defined as mRS scores of 3–6) were similar in the standard blood pressure lowering group (79/116; 68%) and in the individualised blood pressure lowering group (80/126; 63%).^50^ There was no difference in mortality rates (12% vs. 13%).

The Controlling Hypertension and Hypotension Immediately Post Stroke (CHHIPS) included a mixed population of patients with AIS (n = 154) and ICH (n = 25) presenting with systolic blood pressure higher than 160 mm Hg on admission who were treated with labetalol (n = 58), lisinopril (n = 58) or placebo (n = 63). The primary endpoint (death or dependency at 2 weeks) occurred in 14 of 18 patients treated with labetalol or lisinopril (“active treatment”) and 3 of 7 placebo patients in the ICH subgroup. Death occurred in 2 and 0 patients in the active and placebo arms, respectively.^51^

In the subgroup of 629 patients with ICH included in ENOS, GTN was not associated with functional worsening (defined as a 1-point increase across all three-month mRS scores, shift analysis): adjusted common OR: 1.04; 95%CI: 0.78–1.38). There was no association between GTN and three-month mortality (OR: 0.91; 95%CI: 0.55–1.55).^144^ In the subgroup of 61 ICH patients treated within 6 hours, GTN was associated with lower odds of functional worsening at three months (adjusted common OR 0.19; 95% CI 0.06–0.59; p = 0.004) and lower 3-month mortality rates (7% vs. 38%, p = 0.006).^144^

In the subgroup of 145 patients with confirmed ICH included in the RIGHT-2 trial, patients in the GTN group tended to have worse functional outcomes at three months (adjusted common OR for 1-point increase across all three-month mRS scores 1.87, 95%CI: 0.98–3.57). More patients in the GTN died in hospital (adjusted OR 2.26, 95%CI: 1.03–4.95), but there was no difference in death at 90 days (OR 1.50, 95%CI: 0.86–2.62).^29^ Since treatment with GTN was initiated in the ambulance prior to imaging whether these results are due to chance, confounding or the true effect of GTN remains uncertain.

Gupta and co-workers randomised 118 patients with ICH within 72 hours of symptom onset to tight blood pressure control if mean arterial pressure exceeded 115 mm Hg or to conventional blood pressure control if mean arterial pressure ≥130 mm Hg. Patients were randomised within 1 hour of admission and treatment was continued for 72 hours.^145^ There was no difference in the primary endpoint (mRS-scores of 3–6 at 90 days) between the two treatment groups (OR: 0.70; 95%CI: 0.34–1.47).^145^

Koch and co-workers randomised 42 patients with ICH within 8 hours of symptom onset to either aggressive (mean arterial pressure < 110 mm Hg) or standard treatment (mean arterial pressure 110–130 mm Hg).^146^ Treatment duration was 48 hours. The rates of the primary endpoint (neurological deterioration within the first 48 hours) were similar in the aggressive (1/21) and standard (2/21) treatment groups. There were also no differences in 3-month mortality or haematoma expansion (defined as an increase of more than 30% of the initial ICH volume).^146^

In the subgroup analysis of 274 patients with ICH in the SCAST trial, patients in the candesartan group had significantly worse functional outcome (defined as 1-point increase across all six-month mRS scores), adjusted common OR 1.61, 95% CI: 1.03–2.50.^147^

The Intracerebral Haemorrhage Acutely Decreasing Arterial Pressure Trial (ICH-ADAPT) evaluated the influence of systolic blood pressure lowering on cerebral blood flow (CBF). Patients with acute ICH and systolic blood pressure >150 mm Hg on admission, brain CT within 24 hours after symptom onset and no contraindication to CT angiography were randomised to systolic blood pressure target of <150 mm Hg (n = 39) compared to a systolic blood pressure target of <180 mm Hg (n = 36) to be reached within 1 hour of randomisation.^148,149^ At 2 hours, the mean systolic blood pressure was significantly lower in the <150 mm Hg group. However, the primary endpoint (perihaematomal relative CBF) was not different between the two treatment groups. There were no significant differences in mortality rates at day 30: 18% in the <150 mm Hg group and 11% in the <180 mm Hg group, and no difference in functional outcomes at 90 days.^149^

The Perioperative Antihypertensive Treatment in Patients With Spontaneous Intracerebral Haemorrhage (PATICH) was a single centre, assessor-blinded RCT that investigated the effect of peri-operative anti-hypertensive therapy. Adult patients with imaging-proven (brain CT or MRI) acute ICH, elevated systolic blood pressure (150–220 mm Hg) and the need for surgery within 24 hours after onset were randomised within 1 hour of admission to an intensive treatment group (n = 100; systolic blood pressure within the first hour after admission: 140–160 mm Hg, intraoperatively: 120–140 mm Hg, after operation: 120–140 mm Hg for 7 days) or conservative treatment group (n = 101; perioperative systolic blood pressure: 140–180 mm Hg, intraoperatively systolic blood pressure: 90–140 mm Hg).^150^ The primary endpoint was the incidence of re-haemorrhage within 7 days after randomisation “defined as a greater postoperative haematoma volume, a difference of ≤5 mL between the pre- and postoperative haematoma volumes or a difference of ≥5 mL in haematoma volumes between the first postoperative computed tomography and the subsequent computed tomography”. The re-haemorrhage rates did not differ between the intensive (11%) and conservative (14%) groups. The mortality rates at 90 days were also similar.^150^

**Figure 7.**
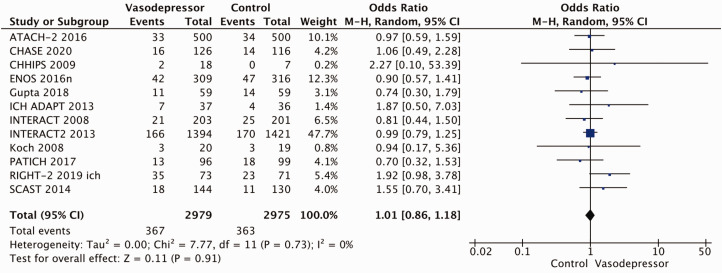
The effect of intensive blood pressure lowering with any vasodepressor drug compared to control on mortality at three to six months following symptom onset in patients with acute intracerebral haemorrhage.

**Figure 8.**
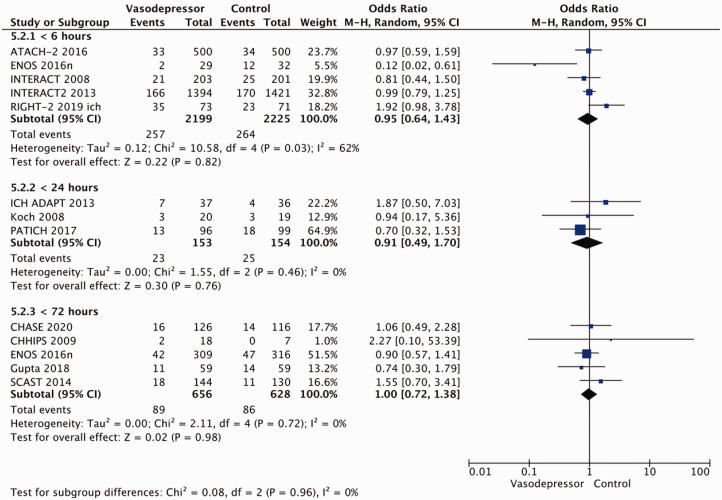
The effect of intensive blood pressure lowering with any vasodepressor drug compared to control on mortality at three to six months following symptom onset in in subgroups stratified by time to treatment (trials enrolling patients within 6 hours, trials enrolling patients within 24 hours after exclusion of trials enrolling patients within 6 hours, and trials enrolling patients within 72 hours after excluding trials enrolling within 24 hours).

Twelve RCTs were included in the meta-analysis of blood pressure lowering with any vasodepressor drug compared with control on mortality at 3-6 months, and there were no differences between the groups (OR 1.01 [95%CI: 0.86, 1.18]; I^2^ = 0%; Figure 7, Table 9).^21,29,37,50,51,141,142,144,145–147,149,150^ Treatment time window varied between 3 and 72 hours. Subgroup analyses of mortality according to time to treatment revealed no significant associations between blood pressure lowering and mortality at three to six months at different time windows (Figure 8).

**Table 9.** Evidence profile table for intensive blood pressure lowering with any vasodepressor drug in patients with acute intracerebral haemorrhage.

Certainty assessment							№ of patients		Effect		Certainty	Importance
№ of studies	Study design	Risk of bias	Inconsistency	Indirectness	Imprecision	Other considerations	Blood pressure lowering with Vasodepressor	Control	Relative (95% CI)	Absolute (95% CI)		
3–6 months mortality												
12	Randomised trials	Not serious	Not serious	Not serious	Serious^a^	Publication bias strongly suspected^b^	367/2979 (12.3%)	363/2975 (12.2%)	OR 1.01 (0.86 to 1.18)	1 More per 1,000 (from 15 fewer to 19 more)	⨁⨁◯◯ Low	Critical
3–6 months mortality <6 hours												
5	Randomised trials	Not serious	Serious^c^	Not serious	Serious^a^	Publication bias strongly suspected^d^	257/2199 (11.7%)	264/2225 (11.9%)	OR 0.95 (0.64 to 1.43)	5 Fewer per 1,000 (from 39 fewer to 43 more)	⨁◯◯◯ Very low	Critical
3–6 months mortality ≤24 hours												
3	Randomised trials	Not serious	Not serious	Not serious	Serious^a^	Publication bias strongly suspected^d^	23/153 (15.0%)	25/154 (16.2%)	OR 0.91 (0.49 to 1.70)	12 fewer per 1,000 (from 76 fewer to 85 more)	⨁⨁◯◯ Low	Critical
3–6 months mortality <72 hours												
5	Randomised trials	Not serious	Not serious	Not serious	Serious^a^	Publication bias strongly suspected^d^	89/656 (13.6%)	86/628 (13.7%)	OR 1.00 (0.72 to 1.38)	0 fewer per 1,000 (from 34 fewer to 43 more)	⨁⨁◯◯ Low	Critical
3–6 months good functional outcome (mRS scores 0–2)												
10	Randomised trials	Not serious	Not serious	Not serious	Serious^a^	None	1285/2894 (44.4%)	1240/2903 (42.7%)	OR 1.05 (0.91 to 1.20)	12 more per 1,000 (from 23 fewer to 45 more)	⨁⨁⨁◯ Moderate	Critical
3–6 months good functional outcome (mRS scores 0–2) – <6 hours												
5	Randomised trials	Not serious	Not serious	Not serious	Serious^a^	Publication bias strongly suspected^d^	998/2169 (46.0%)	964/2196 (43.9%)	OR 1.09 (0.97 to 1.23)	21 More per 1,000 (from 7 fewer to 51 more)	⨁⨁◯◯ Low	Critical
3–6 months good functional outcome (mRS scores 0–2) – <24 hours												
2	Randomised trials	Not serious	Not serious	Not serious	Very serious^e^	Publication bias strongly suspected^d^	37/116 (31.9%)	29/118 (24.6%)	OR 1.20 (0.42 to 3.45)	35 More per 1,000 (from 125 fewer to 283 more)	⨁◯◯◯ Very low	Critical
3–6 months good functional outcome (mRS scores 0–2) – <72 hours												
4	Randomised trials	Not serious	Not serious	Not serious	Serious^a^	Publication bias strongly suspected^d^	260/638 (40.8%)	256/621 (41.2%)	OR 0.97 (0.75 to 1.26)	7 fewer per 1,000 (From 68 fewer to 57 more)	⨁⨁◯◯ Low	Critical
Haematoma expansion												
5	Randomised trials	Not serious	Not serious	Not serious	Serious^a^	Publication bias strongly suspected^d^	254/1173 (21.7%)	279/1128 (24.7%)	OR 0.84 (0.62 to 1.13)	31 Fewer per 1,000 (From 78 fewer to 23 more)	⨁⨁⨁◯ Moderate	Critical
Haematoma expansion – <6 hours												
3	Randomised trials	Not serious	Not serious	Not serious	Not serious	Publication bias strongly suspected^d^	239/1115 (21.4%)	269/1071 (25.1%)	OR 0.81 (0.67 to 0.99)	38 Fewer per 1,000 (from 68 fewer to 2 fewer)	⨁⨁⨁◯ Moderate	Critical
Hematoma expansion – ≤24 hours												
2	Randomised trials	Not serious	Not serious	Not serious	Very serious^e^	Publication bias strongly suspected^d^	15/58 (25.9%)	10/57 (17.5%)	OR 1.66 (0.67 to 4.10)	86 More per 1,000 (From 51 fewer to 290 more)	⨁◯◯◯ Very low	Critical
Acute Renal injury												
4	Randomised trials	Not serious	Not serious	Not serious	Very serious^e^	Publication bias strongly suspected^d^	6/947 (0.6%)	7/932 (0.8%)	OR 0.87 (0.28 to 2.74)	1 Fewer per 1,000 (from 5 fewer to 13 more)	⨁◯◯◯ Very low	Important

CI: confidence interval; OR: odds ratio.

^a^Wide confidence intervals.

^b^Five or less studies reporting this outcome.

^c^Significant heterogeneity, I2 ≥ 62%.

^d^Very wide confidence intervals.

PICO 8: In patients with acute intracerebral haemorrhage, does continuing versus temporarily stopping previous oral antihypertensive therapy improve outcome?

**Figure 9.**
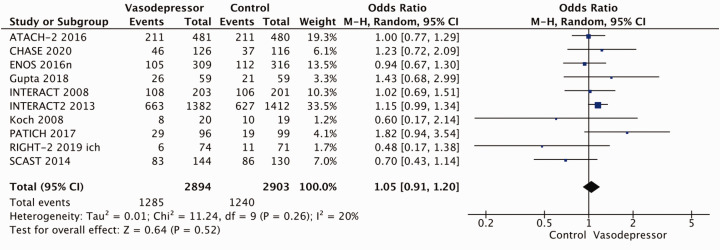
The effect of intensive blood pressure lowering with any vasodepressor drug compared to control on good functional outcome (defined as mRS scores 0–2 at three to six months following symptom onset) in patients with acute intracerebral haemorrhage.

**Figure 10.**
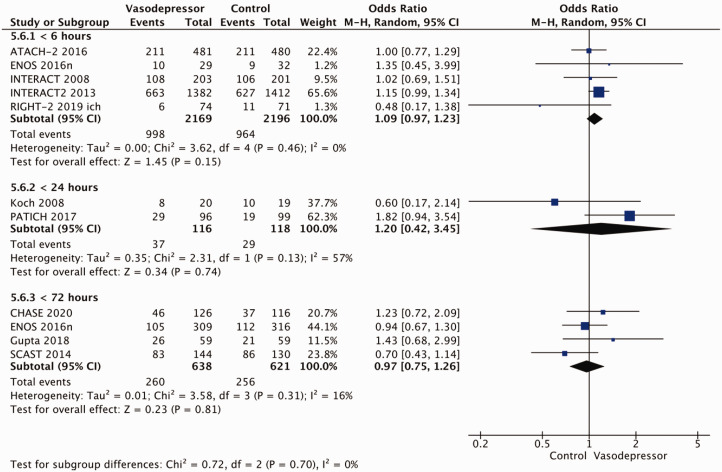
The effect of intensive blood pressure lowering with any vasodepressor drug compared to control on good functional outcome (defined as mRS scores 0–2 at three to six months following symptom onset) in subgroups stratified by time to treatment (trials enrolling patients within 6 hours, trials enrolling patients within 24 hours after exclusion of trials enrolling patients within 6 hours, and trials enrolling patients within 72 hours after excluding trials enrolling within 24 hours).

Data on good functional outcome defined as mRS scores of 0–2 at three to six months were available from 10 RCTs.^21,29,50,141,142,144-147,150^ Blood pressure lowering with any vasodepressor was associated with no benefit on good functional outcome (OR 1.05, 95%CI: 0.91–1.20; I^2^ = 20%; Figure 9, Table 9) Subgroup analyses of good functional outcome (mRS scores 0–2) at three to six months according to treatment time window revealed no significant associations between blood pressure lowering and good functional outcomes at three to six months at different time windows (Figure 10, Table 9).

**Figure 11.**
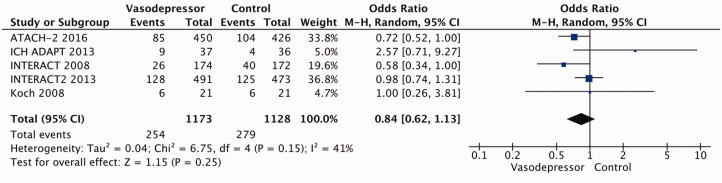
The effect of intensive blood pressure lowering with any vasodepressor drug compared to control on haematoma expansion.

**Figure 12.**
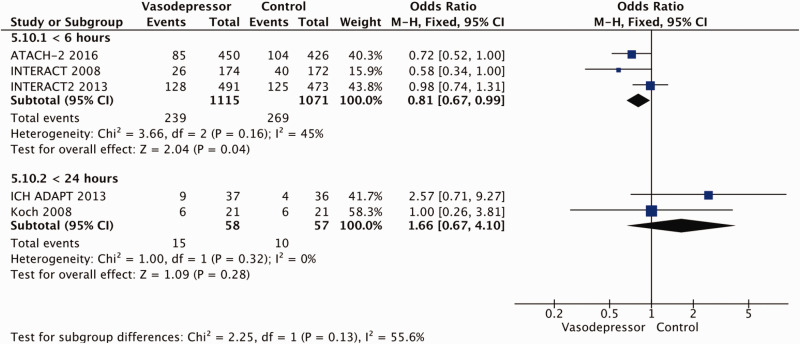
The effect of intensive blood pressure lowering with any vasodepressor drug compared to control on haematoma expansion in subgroups stratified by time to treatment (trials enrolling patients within 6 hours, trials enrolling patients within 24 hours after exclusion of trials enrolling patients within 6 hours).

Data on haematoma expansion were available from 5 RCTs.^21,141,142,146,149^ Overall, intensive blood pressure lowering treatment did not reduce haematoma expansion (RR = 0.84, 95%CI:0.62–1.13; I^2^ = 41% Figure 11, Table 9). In the subgroup analysis of trials randomising ICH patients within 6 hours from symptom onset (Figure 12, Table 9) intensive blood pressure lowering was associated with lower likelihood of haematoma expansion: OR = 0.81;95%CI: 0.67–0.99; I^2^ = 45%. There was no marked effect of antihypertensive treatment when time treatment window was ≤24 hours (Figure 12). These data support a potential biological effect of blood pressure lowering therapy on haematoma expansion.

Acute renal injury has been described as serious adverse event of intensive blood pressure lowering in spontaneous ICH. Analysis of the four trials reported that acute renal injury rates in the treatment arms revealed no association between intensive blood pressure lowering and acute renal injury in ICH (OR 0.87; 95%CI: 0.28–2.74, Table 9).

Table 9 provides the evidence profile regarding the safety and efficacy of intensive blood pressure lowering in acute ICH. The existing data is of moderate quality and predominantly concerns conscious patients, who do not require surgical intervention. Furthermore, there are limited data on patients treated very early from ICH onset (minutes to hours). Finally, it should be noted that ATACH-II excluded patients with haematoma volumes >60 cm^3^,^142^ while INTERACT-2 excluded patients if “they had a massive haematoma with a poor prognosis, or if early surgery to evacuate the haematoma was planned”.^21^ Thus, the presented associations predominantly correspond to acute ICH patients with small to moderate haematoma volumes.

### Additional information

Burgess and co-workers performed a prospective cohort study on 448 patients with acute spontaneous ICH and looked at the effect of the extent of blood lowering on the occurrence of acute renal injury in patient with and without chronic kidney disease.^151^ The risk of acute renal injury was associated with >90 mm Hg reduction of baseline systolic blood pressure.

In order to achieve rapid and controlled blood pressure lowering, drugs used need to be fast-acting with a short half-life time to reduce the risk of excessive blood pressure lowering. Most of the currently used antihypertensives (urapidil, labetalol, esmolol, nicardipine) do only partially fulfil these criteria. Clevidipine is a calcium channel antagonist with a half-life time of 1.5 minutes that may be effective in blood pressure control of patients with acute ICH. Graffagnino and co-workers published a very small prospective case-series of 35 patients with acute ICH treated with clevidipine.^152^ Mean time to target pressure between 140 and 160 mm Hg was 5.5 minutes and mean ICH volume increase was negligible (0,01 ml).

In conclusion, early (<6 hours from symptom) lowering of blood pressure leads to a reduction of haematoma expansion in acute ICH patients. Haematoma expansion is strongly and independently associated with adverse functional outcome at three months in acute ICH.^153^ The question remains: Why does reduction of haematoma expansion not translate into a clinical benefit? Our hypothesis is as follows: The clinical benefit in ICH is primarily derived by preventing lesion increase ( = haematoma expansion). An individual patient data meta-analysis of 5435 acute ICH patients identified two strong predictors of haematoma expansion. First, the time from onset to first brain CT and second, the volume of baseline ICH.^140^ Most haematoma expansion occurred within the first three hours after onset of ICH. The probability of haematoma expansion increased with the volume of the initial lesion up to 75 ml.^140^ In the five RCTs on blood pressure lowering for hyperacute ICH, the mean time from ICH onset to treatment in was 5.7 hours. This timeframe is at least 2 hours longer than the time period where haematoma expansion most commonly occurs.^21^,^141^,^142^,^146^,^149^

The mean volume of the initial lesion in the five RCTs was 14 ml, which may be too small to show a clinically meaningful change by preventing haematoma expansion.^21^,^141^,^142^,^146^,^149^ Since the evidence available is primarily from patients with small haematoma volume, uncertainty remains regarding the effect of blood pressure lowering in patients with large initial haematoma volumes. Also, it should be noted that in addition to haematoma expansion there are other factors impacting 3-month functional outcome in acute ICH including neurocritical care, location of baseline haematoma volume, end-of-life health policies across different countries and these factors may have moderated or diluted the potential association of early (<6 h) intensive blood pressure lowering and functional outcome at three months.

A robust marker of ongoing bleeding, studied extensively over the past years, is the CT angiography spot sign.^153,154^ The spot sign is commonly assumed to represent continued bleeding (e.g., contrast extravasation visualised on CTA after contrast bolus injection) from ruptured vessels surrounding the initial haematoma. The spot sign has been associated with both haematoma expansion and poor clinical outcomes in acute ICH patients.^155^ SCORE-IT (Spot Sign Score in Restricting ICH Growth) is a preplanned prospective observational study nested in the ATACH-II RCT including consecutive patients with primary ICH who underwent a CT angiography within 8 hours from onset.^156^ A total of 133 patients were included in this preplanned analysis. Of these, 40% had a spot sign, and 20% experienced haematoma expansion. After adjustment for potential confounders, intensive blood pressure treatment was not associated with a significant reduction of haematoma expansion (relative risk: 0.83; 95%CI: 0.27–2.51; p* = *0.74) or functional outcome (relative risk of 90-day mRS score ≥4, 1.24; 95%CI: 0.53–2.91; p = 0.62) in spot sign–positive patients.


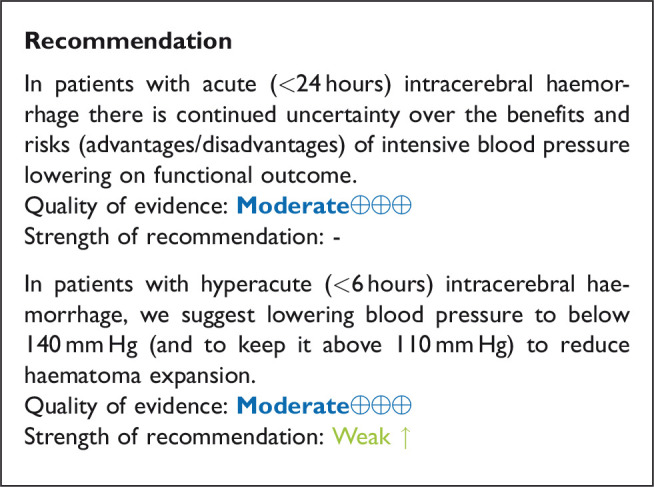

Expert consensus statement

In patients with acute intracerebral haemorrhage, we suggest initiating antihypertensive treatment as early as possible and ideally within 2 hours of symptom onset. The decrease of systolic blood pressure should not exceed 90 mm Hg from baseline values. Vote 10 of 10.

In patients with acute intracerebral haemorrhage, we suggest lowering blood pressure according to recommended levels beyond 6 hours after onset of treatment for at least 24 hours and up to 72 hours to reduce haematoma expansion. Vote 10 of 10.

## In patients with acute intracerebral haemorrhage, does continuing versus temporarily stopping previous oral antihypertensive therapy improve outcome?

### Rationale

Blood pressure lowering therapy is a key strategy for the primary and secondary prevention of ICH and other serious cardiovascular events. However, it is unclear if it is better to continue or temporarily stop prior ongoing antihypertensive agent(s) in the setting of an acute ICH. One theoretical argument in favour of continuing blood pressure lowering therapy in acute ICH is related to more effective blood pressure control that in turn may limit haematoma expansion. On the other hand, it may be argued that continuing oral antihypertensive agents may worsen functional outcome due to compromise in cerebral blood flow and perihaematomal perfusion; furthermore, it may increase the risk of aspiration pneumonia.

#### Analysis of current evidence

One RCT, ENOS, included 246 patients with acute ICH and elevated systolic blood pressure (140–220 mm Hg) who were previously on antihypertensive therapy. The patients were randomised to continue or stop previous blood pressure lowering treatment for seven days.^157^ There was no difference in mRS scores between the treatment groups at day 90 (common OR for worse functional outcome, defined by 1-point increase across all mRS grades in the group that continued blood pressure lowering treatment 0.92; 95% CI: 0.45–1.89; p = 0.83). There was no significant difference in mortality between the 2 groups at day 90 (16.0% and 18.3% in the continue versus stop groups, respectively).^157^ There was no information on haematoma expansion.

**Figure 13.**
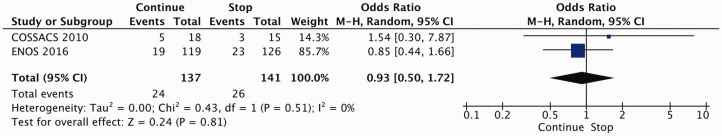
The effect of continuing versus temporarily stopping previous blood pressure lowering therapy on mortality at three to six months following symptom onset in patients with acute intracerebral haemorrhage.

**Figure 14.**
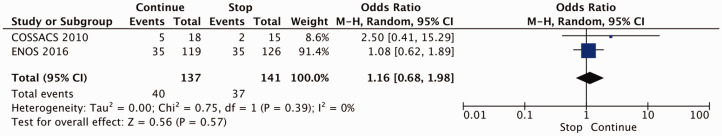
The effect of continuing versus temporarily stopping previous blood pressure lowering therapy on good functional outcome (defined as mRS scores 0–2) at three to six months following symptom onset in patients with acute intracerebral haemorrhage.

A meta-analysis of individual patient data from COSSACS and the ENOS trials evaluated the effect of continuing versus stopping temporarily previous blood pressure-lowering therapy on death or dependency in 2860 patients with acute stroke.^139^ The meta-analysis indicated no significant association of continuing versus stopping previous blood pressure lowering therapy with the odds of death or the improved functional outcome in the subgroup of patients with ICH (Table 10).^139^ Two trials^37^,^138^ were included in the meta-analysis on continuing versus stopping previous blood pressure lowering therapy. There were no significant differences between the continuing versus stopping previous blood pressure lowering treatment on mortality (OR 0.93, 95% CI 0.50 – 1.72, p = 0.81, I^2^ = 0%; Figure 13) nor on good functional outcome (mRS 0–2) (OR 1.16, 95 % CI 0.68 – 1.98, p = 0.57, I^2^ = 0%; Figure 14).

**Table 10.** Evidence profile table for continuing versus temporarily stopping previous blood-pressure lowering therapy in patients with acute intracerebral hemorrhage.

Certainty assessment							№ of patients		Effect		Certainty	Importance
№ of studies	Study design	Risk of bias	Inconsistency	Indirectness	Imprecision	Other considerations	Continuing	Stopping previous antihypertensive therapy	Relative (95% CI)	Absolute (95% CI)		
3–6 months mortality												
2	Randomised trials	Not serious	Not serious	Not serious	Serious^a^	Publication bias strongly suspected^b^	24/137 (17.5%)	26/141 (18.4%)	OR 0.93 (0.50 to 1.72)	11 Fewer per 1,000 (from 83 fewer to 96 more)	⨁⨁◯◯ Low	Critical
3–6 month good functional outcome (mRS scores 0–2)												
2	Randomised trials	Not serious	Not serious	Not serious	Serious^a^	Publication bias strongly suspected^b^	40/137 (29.2%)	37/141 (26.2%)	OR 1.16 (0.68 to 1.98)	30 More per 1,000 (From 68 fewer to 151 more)	⨁⨁◯◯ Low	Critical

CI: confidence interval; OR: odds ratio.

^a^Wide confidence intervals.

^b^Two studies reported this outcome.

Table 10 provides details regarding the safety and efficacy of continuing versus temporarily stopping previous blood-pressure lowering therapy in patients with acute ICH based on published and unpublished data from the individual patient data meta-analysis.^139^

### Additional information

When deciding to continue or stop temporarily previous antihypertensive agents individual blood pressure levels of ICH patients and the need to use or not to use antihypertensive agents to maintain these levels within the range recommended for patients with acute ICH needs to be considered. The most common situation in clinical practice is the need of a blood pressure lowering therapy to maintain blood pressure levels within the recommended range. In this case continuation of previous antihypertensive agents may favour more stable blood pressure control. However, the continuation of oral antihypertensive agents may be challenging in patients with dysphagia and impaired consciousness. The route of application depends on the ability to swallow and/or the degree of consciousness.


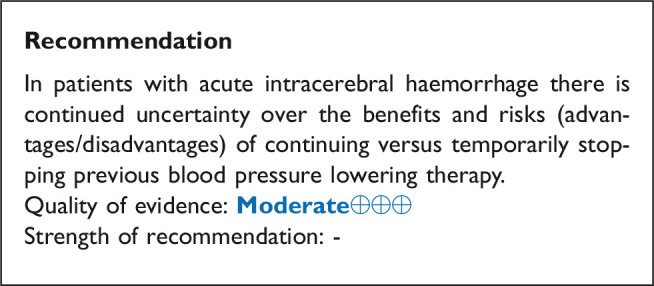

Expert consensus statement

In patients acute intracerebral haemorrhage who need blood pressure lowering therapy to maintain blood pressure within the recommended range and who do not have swallowing problems, we suggest continuation of prior oral antihypertensive agents. Vote 10 of 10.

In patients with acute intracerebral haemorrhage who need blood pressure lowering therapy to maintain blood pressure within the recommended range and who have dysphagia or decreased level of consciousness, we suggest temporarily stopping previous oral hypertensive therapy and using intravenous antihypertensive agents until swallowing is restored or a nasogastric tube is in place. Vote 10 of 10.

## Discussion

This guideline document was developed using the GRADE methodology and aims to assist physicians in the management of blood pressure in AIS and ICH. All recommendations and Expert consensus statements are summarised in Table 11.

**Table 11.** Evidence table for ESO Guidelines on blood pressure management in acute ischaemic stroke and intracerebral haemorrhage

PICO Question	Recommendations	Expert consensus statement
PICO 1: In patients with suspected acute stroke, does pre-hospital blood pressure lowering with any vasodepressor drug compared to no drug improve outcome?	In patients with suspected stroke we suggest against routine blood pressure lowering in the pre-hospital setting. Quality of evidence: Moderate⊕⊕⊕Strength of recommendation: Weak ↓?	Due to the potential harm in patients with intracerebral haemorrhage prehospital treatment with glyceryl trinitrate should be avoided. Vote 9 of 10.

PICO 2: In hospitalised patients with acute ischaemic stroke not treated with reperfusion therapies (intravenous thrombolysis or mechanical thrombectomy), does blood pressure lowering with any vasodepressor drug compared to no drug improve outcome?	In hospitalised patients with acute ischaemic stroke and blood pressure < 220/110 mmHg not treated with intravenous thrombolysis or mechanical thrombectomy, we suggest against the routine use of blood pressure lowering agents at least in first 24 hours following symptom onset, unless this is necessary for a specific comorbid condition. Quality of evidence: Moderate⊕⊕⊕ Strength of recommendation: Weak ↓?	In patients with acute ischaemic stroke not treated with intravenous thrombolysis or mechanical thrombectomy and blood pressure >220/120 mmHg, careful blood pressure reduction (<15% systolic blood pressure reduction in 24 hours) is reasonable and likely to be safe. No specific blood pressure lowering agent can be recommended. Vote 10 of 10.

PICO 3: In hospitalised patients with acute ischaemic stroke and undergoing intravenous thrombolysis (with or without mechanical thrombectomy), does blood lowering with any vasodepressor drug compared to control improve outcome?	In patients with acute ischaemic stroke undergoing treatment with intravenous thrombolysis (with or without mechanical thrombectomy) we suggest maintaining blood pressure below 185/110 mmHg before bolus and below 180/105 mmHg after bolus, and for 24 hours after alteplase infusion. No specific blood pressure-lowering agent can be recommended. Quality of evidence: Very low⊕ Strength of recommendation: Weak ↑? In patients with acute ischaemic stroke undergoing treatment with intravenous thrombolysis (with or without mechanical thrombectomy) we suggest against lowering systolic blood pressure to a target of 130-140mmHg compared to <180mmHg during the first 72 hours following of symptom onset. Quality of evidence: Moderate⊕⊕⊕ Strength of recommendation: Weak ↓?	

PICO 4: In patients with acute ischaemic stroke caused by large vessel occlusion and undergoing mechanical thrombectomy (with or without intravenous thrombolysis), does blood pressure lowering with any vasodepressor drug compared to no drug improve outcome?	In patients with acute ischaemic stroke due to large vessel occlusion undergoing mechanical thrombectomy (with or without intravenous thrombolysis) we suggest keeping blood pressure below 180/105 mmHg during, and 24 hours after, mechanical thrombectomy. No specific blood pressure-lowering agent can be recommended. Quality of evidence: Very low⊕ Strength of recommendation: Weak ↑? In patients with acute ischaemic stroke due to large vessel occlusion we suggest against actively reducing systolic blood pressure <130mmHg during the first 24 hours following successful mechanical thrombectomy Quality of evidence: Moderate⊕⊕⊕ Strength of recommendation: Weak ↓? In patients with acute ischaemic stroke due to large vessel occlusion undergoing treatment with mechanical thrombectomy (with or without intravenous thrombolysis) systolic blood pressure drops should be avoided. Quality of evidence: Very low⊕ Strength of recommendation: Strong ↓↓	In patients with acute ischaemic stroke due to large vessel occlusion who achieve successful reperfusion defined as modified Thrombolysis in Cerebral Infarction grade of 3 following mechanical thrombectomy we suggest against induced hypertension. Vote 10 of 10

PICO 5: In patients with acute ischaemic stroke not treated with reperfusion therapies (intravenous thrombolysis or mechanical thrombectomy) and with clinical deterioration, does induced hypertension by any vasopressor drug compared to no drug improve outcome?	In patients with acute ischaemic stroke not treated with reperfusion therapies (intravenous thrombolysis or mechanical thrombectomy) who experience clinical deterioration, we suggest against the routine use of vasopressor drugs to increase blood pressure. Quality of evidence: Very low ⊕ Strength of recommendation: Weak ↓↓	In patients with acute ischaemic stroke not treated with reperfusion therapies (intravenous thrombolysis or mechanical thrombectomy) and with clinical deterioration where a haemodynamic mechanism is suspected or shown to be directly responsible for the deterioration, we suggest: • stopping existing blood pressure lowering therapy, • administering intravenous fluids and • introducing non-pharmacological procedures to raise blood pressure before considering • careful use of vasopressor agents to increase blood pressure with close monitoring of blood pressure values. Vote 10 of 10.

PICO 6: In patients with acute ischaemic stroke, does continuing versus temporarily stopping previous oral blood pressure lowering therapy improve outcome?	In patients with acute ischaemic stroke, there is continued uncertainty over the benefits and risks (advantages/disadvantages) of continuing versus temporarily stopping previous blood pressure lowering therapy. Quality of evidence: Moderate⊕⊕⊕ Strength of recommendation: -	In patients with acute ischaemic stroke we suggest stopping previous oral blood pressure lowering therapy in patients with dysphagia until swallowing is restored or a nasogastric tube is in place. Vote 10 of 10

PICO 7: In patients with acute intracerebral haemorrhage, does intensive blood pressure lowering with any vasodepressor drug compared to control improve outcome?	In patients with acute (<24 hours) intracerebral haemorrhage there is continued uncertainty over the benefits and risks (advantages/disadvantages) of intensive blood pressure lowering on functional outcome. Quality of evidence: Moderate⊕⊕⊕ Strength of recommendation: - In patients with hyperacute (<6 hours) intracerebral haemorrhage, we suggest lowering blood pressure to below 140 mmHg (and to keep it above 110 mmHg) to reduce haematoma expansion. Quality of evidence: Moderate⊕⊕⊕ Strength of recommendation: Weak ↑	In patients with acute intracerebral haemorrhage, we suggest initiating antihypertensive treatment as early as possible and ideally within 2 hours of symptom onset. The decrease of systolic blood pressure should not exceed 90 mmHg from baseline values. Vote 10 of 10. In patients with acute intracerebral haemorrhage, we suggest lowering blood pressure according to recommended levels beyond 6 hours after onset of treatment for at least 24 hours and up to 72 hours to reduce haematoma expansion. Vote 10 of 10.

PICO 8: In patients with acute intracerebral haemorrhage, does continuing versus temporarily stopping previous oral antihypertensive therapy improve outcome?	In patients with acute intracerebral haemorrhage there is continued uncertainty over the benefits and risks (advantages/disadvantages) of continuing versus temporarily stopping previous blood pressure lowering therapy. Quality of evidence: Moderate⊕⊕⊕ Strength of recommendation: -	In patients acute intracerebral haemorrhage who need blood pressure lowering therapy to maintain blood pressure within the recommended range and who do not have swallowing problems, we suggest continuation of prior oral antihypertensive agents. Vote 10 of 10. In patients with acute intracerebral haemorrhage who need blood pressure lowering therapy to maintain blood pressure within the recommended range and who have dysphagia or decreased level of consciousness, we suggest temporarily stopping previous oral hypertensive therapy and using intravenous antihypertensive agents until swallowing is restored or a nasogastric tube is in place. Vote 10 of 10.

We have based all our recommendations on RCTs or individual patient data meta-analyses whenever possible, but for certain PICO questions most of the evidence is based on observational studies, with the limitations this type of evidence entails. We provide analyses in AIS and in ICH subgroups separately. Although many of the trials covered in our guideline enrolled patients with both ischaemic and haemorrhagic stroke subtypes, we believe that it is appropriate to generate separate recommendations for these two subtypes due to the differences in the pathophysiology. In the AIS subgroup we have made specific recommendations for patients undergoing acute reperfusion therapies with intravenous alteplase, and/or endovascular treatment. These are two specific settings where discussions regarding appropriate blood pressure management arise in most acute stroke departments daily. The use of vasopressors is increasing in the management of low blood pressure in AIS, especially in patients undergoing endovascular procedure where large drops in blood pressure is detrimental and in patients with fluctuating symptoms with an assumed haemodynamic aetiology. We therefore provided specific recommendations for these additional settings. For ICH we further classified our recommendations on blood pressure management according to thresholds, timing and agent/strategy.

The strengths of the current guideline are the systematic search of the literature and use of the GRADE methodology to provide guidance for clinicians. Our group conducted multiple analyses of the available data using a strict meta-analytical approach. Nevertheless, despite making our recommendations based on randomised controlled data in different stroke subgroups, the level of evidence is consistently very low, low or moderate. This exemplifies the complexity of blood pressure management in acute stroke settings where epidemiological evidence, pathophysiological evidence and data from RCTs are conflicting. This also likely reflects the heterogeneity of the trials included regarding timing of blood pressure management, inclusion criteria and blood pressure lowering strategies. To provide guidance for clinical practice we made nine expert consensus statements in addition to the official recommendations due to the equipoise in clinical evidence. These statements have been voted on among the working group. All the statements had excellent agreement (10/10 in 8 statement and 9/10 in one statement) among the group members.

Many questions regarding blood pressure management in acute stroke remain unanswered. First, in both patients with AIS and ICH blood pressure is a highly individual parameter and the association between blood pressure and outcome is likely to be multifactorial. The “one-target fits all” may not be appropriate in acute stroke settings, and future trials should consider premorbid hypertension, baseline blood pressure values and relative reductions in baseline blood pressure rather than arbitrary absolute blood pressure targets. As shown in the evidence presented in these guidelines, the detrimental effect of high blood pressure on both short- and long-term outcome in AIS is not altered by blood pressure lowering. There are also multiple stroke related factors contributing to elevated blood pressure levels in AIS including ischaemic stroke subtype, recanalisation status and collateral flow that should be considered in the design of future trials. In addition, future trials should differentiate between the different blood pressure management strategies before, during and after successful reperfusion evaluating personalised autoregulation-oriented blood pressure thresholds.

For patients with acute ICH we have shown effects of blood pressure lowering on haematoma growth within a narrow time window of 6 hours but not on functional outcome. Overall, haematoma size in the patients included in the trials was small and treatment was in some of the trials initiated treatment up to 48 hours after symptom onset, which is likely too late to influence outcome. There is also the possibility that manipulating blood pressure is not effective enough alone to influence functional outcome but may work synergistically as part of a bundle of care that can carry a greater impact on functional outcome. Future trials should investigate the efficacy of blood pressure lowering therapies in the hyperacute time window of first 6 hours following symptom onset in patients with acute ICH and evaluate blood pressure lowering agents that reduce blood pressure variability in addition to absolute blood pressure levels.

## Plain language summary

The majority of patients with stroke either due to blood clots (ischaemic stroke) or brain bleeds (intracerebral haemorrhage) have high blood pressure both in the ambulance and on admission to the hospital. We know that high blood pressure in the acute phase of stroke can lead to death, new strokes and poor functional outcome. Despite this, we don’t know whether we should treat high blood pressure as the damaged brain might need higher blood pressure to ensure adequate blood flow. The guideline authors provide ten recommendation, the most important being:

1. Blood pressure in patients with suspected stroke should not be treated in the ambulance
2. If the stroke is due to a blood clot in the arteries:
  - Blood pressure should not be treated unless the blood pressure is very high (systolic blood pressure >220 mm Hg) or if the patients can receive treatment with clot-busting drugs (thrombolytics) or clot-fishing (endovascular treatment).
  - Giving drugs to increase blood pressure should be avoided in the majority of patients
  - If the patient is already under treatment with blood pressure lowering drugs at the time of admission these should be stopped in patients who cannot swallow
3. If the stroke is caused by a brain bleed:
  - Elevated blood pressure should be treated as early as possible after symptom onset
  - If patients are already under treatment with blood pressure drugs these should be continued during the hospital stay

## Supplemental Material

sj-pdf-1-eso-10.1177_23969873211012133 - Supplemental material for European Stroke Organisation (ESO) guidelines on blood pressure management in acute ischaemic stroke and intracerebral haemorrhage Click here for additional data file. Supplemental material, sj-pdf-1-eso-10.1177_23969873211012133 for European Stroke Organisation (ESO) guidelines on blood pressure management in acute ischaemic stroke and intracerebral haemorrhage by Else Charlotte Sandset, Craig S Anderson, Philip M Bath, Hanne Christensen, Urs Fischer, Dariusz Gąsecki, Avtar Lal, Lisa S Manning, Simona Sacco, Thorsten Steiner and Georgios Tsivgoulis in European Stroke Journal
