# Supplementary material for: European Stroke Organisation (ESO) guidelines on blood pressure management in acute ischaemic stroke and intracerebral haemorrhage
Source: Eur Stroke J. 2021 May 11;6(2):XLVIII–LXXXIX. doi: 10.1177/23969873211012133 (PMC8370078; doi:10.1177/23969873211012133)

European Stroke Organisation (ESO) Guidelines on Blood Pressure Management in Acute Ischaemic Stroke and Intracerebral Haemorrhage

Supplemental material

METHODS

This systematic review and meta-analysis followed the guidelines of ESO Standard Operating Procedures. A comprehensive search of literature was performed on the role of various interventions in patients with unruptured aneurysm. Databases such as MEDLINE, EMBASE, CINAHL registers were employed for the literature search. Following search terms and their corresponding Medical Subject Heading (MeSH) terms were used to identify the articles and these searches varied with the PICOs.

Only English language articles were included, with no minimum search date through September 30, 2020. A hand search of some systematic reviews and guidelines was performed and experts were contacted for the existence of both unpublished and published studies that we were not aware of.

Study Selection

The studies were imported into the Covidence software (Covidence systematic review software, Veritas Health Innovation, Melbourne, Australia) and duplicate articles were removed. Two assessors performed a "First level selection" or "Titles and abstracts screening" by following the inclusion/ exclusion criteria (see below) independently and in duplicate. These criteria varied with the PICO

A study was included if it reported the components of predetermined inclusion criteria. The inclusion criteria varied with the PICO. The study was excluded if it had one or more exclusion reasons or did not consider the components of inclusion criteria. A study may be considered potentially relevant if few components of the inclusion criteria are reported in the title or abstract or there was insufficient information to exclude the study. Full texts of all the included or potentially relevant articles were loaded into Covidence software. "Second level selection" or "Full text screening" of these articles was performed by reading the full text of the articles selected at "First level selection" and following the same inclusion/ exclusion criteria. Second level selection was performed in duplicate by two assessors independently. Discrepancies or conflicts in selection or rejection of studies were resolved by consensus or by a third reviewer when needed. This was done both at the first and second level selection.

In studies with duplicate data (companion publications), the original study or the study reporting detailed or recent data (with a greater number of subjects) was included. When different outcomes were reported in the studies, all of these were included.

Data Extraction

Data extraction of outcomes was performed by one reviewer and checked by another reviewer.

Risk of Bias Assessment

The risk of bias of RCTs was assessed using the COCHRANE Collaboration's tool. We assessed randomization (random sequence generation), allocation concealment, blinding of participants, outcome assessment, attrition bias (incomplete outcome data), reporting bias (selective reporting) and other biases in each study.

Meta-analysis

Meta-analysis was performed using Review Manager (RevMan) 5.3 COCHRANE Collaboration software when more than one study reported the outcome and number of subjects were ≥ 6 in each group. Odds ratios and 95% CI for dichotomous variables were calculated. I^2 statistic, an expression of inconsistency of studies' results and describing the percentage of variation across studies due to heterogeneity rather than by chance, was calculated. A high value of I^2 (>50%) and p value <0.05 indicate statistically significant heterogeneity among the studies for an outcome. The reasons for high heterogeneity were explored. A random effects model was used for all outcomes. Where appropriate, subgroup analyses based on age of the patients, time to intervention were performed.

GRADE

The evidence was assessed using the GRADE process that considered the following components of GRADE: study design, risk of bias, heterogeneity, directness, precision, and publication bias, as well as magnitude of effect, confounding, and dose response relationship. GRADE pro software was used and based on addressing the components of GRADE, the evidence was graded as high, moderate, low, or very low quality.

Guyatt GH, Oxman AD, Schünemann HJ, Tugwell P, Knottnerus A. GRADE guidelines: a new series of articles in the Journal of Clinical Epidemiology. J Clin Epidemiol. 2011;64(4):380-2.

Ntaios G, Bornstein NM, Caso V, Christensen H, De Keyser J, Diener HC, et al. The European Stroke Organisation Guidelines: a standard operating procedure. Int J Stroke. 2015;10 Suppl A100:128-35.

SEARCH STRATEGY

PICO 1: In patients with suspected acute stroke, does pre-hospital blood pressure lowering with any vasodepressor drug compared to no drug improve outcome?

("stroke" OR "cerebrovascular accident*" OR "CVA" OR "cerebrovascular apoplexy" OR "cerebrovascular infarct*" OR "cerebrovascular embolism" OR "cerebrovascular disorder*" OR "brain ischemia" OR "brain infarct*" OR "brain haemorrhagic stroke" OR "ischaemic stroke" OR "cerebral embolism" OR "cerebral haemorrhage" OR "intracerebral haemorrhage" OR "ICH" OR "cardioembolic stroke" OR "hemiparesis" OR "hemiplegia" OR "Intracranial arteriosclerosis") AND ("blood pressure lowering drug*" OR "blood pressure lowering agent*" OR "antihypertensive*" OR "antihypertensive drug*" OR "beta blockers" OR "atenolol" OR "propanalol" OR "calcium channel blockers" OR "nimodipine" OR "nicardipine" OR "amilodipine" OR "felodipine" OR "isradipine" OR "nifedipine" OR "nisolodipine" OR "angiotensin-converting enzyme inhibitors" OR "ACE inhibitors" OR "captopril" OR "enalapril" OR "lisinopril" OR "perindopril" OR "Ramipril" OR "angiotensin receptor blocker*" OR "angiotensin receptor antagonist*" OR "candesartan" OR "losartan" OR "Telmisartan" OR "valsartan" OR "clonidine" OR "thiazide" OR "bendrofluazide" OR "Bendroflumethiazide" OR

“hydrochlorthiazide” OR “HCT” OR “vasoactive” OR “nitrate” OR “glyceryl trinitrate” OR “GTN” OR “nitric Oxide Donors”) AND (“Prehospital*” OR “pre-hospital*” OR “ambulance” OR “paramedic*” OR “emergency medical service*” OR “EMS” OR “Emergency Medicine Technician*” OR “Emergency Medical Technician*” OR “Emergency adj3 Unit*”)

PICO 2: In hospitalised patients with acute ischaemic stroke not treated with reperfusion therapies (intravenous thrombolysis or mechanical thrombectomy), does blood pressure lowering with any vasodepressor drug compared to no drug improve outcome?

(“ischaemic stroke” OR “Intracranial arteriosclerosis” OR “brain ischemia” OR “brain infarct*” OR “cerebral embolism” OR “Intracranial arteriosclerosis”) AND (“Thrombolysis” OR “thrombolytic” OR “fibrinolysis” OR “fibrinolytic*” OR “fibrinolytic agent*” OR “urokinase” OR “anistreplase” OR “streptokinase” OR “tPA” OR “plasminogen activator*” OR “tissue plasminogen activator*” OR “recombinant tissue plasminogen activator*” OR “rtPA” OR “rt-PA” OR “alteplase” OR “reteplase” OR “tenecteplase” OR “recombinant protein*” OR “endovascular therapy” OR “endovascular treatment” OR “endovascular procedure*” OR “endovascular method*” OR “endovascular stenting” OR “endovascular adverse effect*” OR “endovascular thrombectomy” OR “endovascular embolization” OR “intra-arterial thrombolysis” OR “intraarterial thrombolysis” OR “intraarterial embolectomy” OR “intra-arterial thrombectomy” OR “intraarterial thrombectomy” OR “mechanical thrombolysis” OR “rheolytic thrombectomy” OR “Angiojet” OR “stent type retriever” OR “stent” OR “Trevo Retriever” OR “Penumbra device” OR “Solitaire revascularization Device” OR “balloon angioplasty”) AND (“Blood pressure decrease” OR “blood pressure reduction” OR “blood pressure lowering drugs” OR “antihypertensive*” OR “antihypertensive drug*” OR “beta blockers” OR “atenolol” OR “propanalol” OR “calcium channel blockers” OR “nimodipine” OR “nicardipine” OR “amilodipine” OR “felodipine” OR “isradipine” OR “nifedipine” OR “nisolodipine” OR “angiotensin-converting enzyme inhibitors” OR “ACE inhibitors” OR “captopril” OR “enalapril” OR “lisinopril” OR “perindopril” OR “Ramipril” OR “angiotensin receptor blocker*” OR “angiotensin receptor antagonist*” OR “candesartan” OR “losartan” OR “Telmisartan” OR “valsartan” OR “clonidine” OR “thiazide” OR “bendrofluazide” OR “Bendroflumethiazide” OR “hydrochrlothiazide” OR “HCT” OR “vasoactive” OR “nitrate” OR “glyceryl trinitrate” OR “GTN” OR “nitric Oxide Donors”) AND (“Randomized Controlled Trials” OR “random allocation” OR “Controlled Clinical Trials” OR “control groups” OR “clinical trials” OR “phase I” OR “phase II” OR “phase III” OR “phase IV” OR “double-blind method” OR “single-blind method” OR “randomized controlled trial” OR “controlled clinical trial” OR “random*” OR “RCT*” OR “quasi-random*” OR “pseudo-random*”)

PICO 3: In hospitalised patients with acute ischaemic stroke and undergoing intravenous thrombolysis (with or without mechanical thrombectomy), does blood lowering therapies compared to control improve outcome?

PICO 4: In patients with acute ischaemic stroke caused by large vessel occlusion and undergoing mechanical thrombectomy (with or without intravenous thrombolysis), does

blood pressure lowering with any vasodepressor drug compared to no drug improve outcome?

(“ischaemic stroke” OR “ischemic stroke” OR “Intracranial Arteriosclerosis” OR “brain ischemia” OR “brain infarct*” OR “cerebral embolism” OR “Intracranial arteriosclerosis”) AND (“Thrombolysis” OR “thrombolytic” OR “fibrinolysis” OR “fibrinolytic*” OR “fibrinolytic agent*” OR “urokinase” OR “anistreplase” OR “streptokinase” OR “tPA” OR “plasminogen activator*” OR “tissue plasminogen activator*” OR “recombinant tissue plasminogen activator*” OR “rtPA” OR “rt-PA” OR “alteplase” OR “reteplase” OR “tenecteplase” OR “recombinant protein*” OR “endovascular therapy” OR “endovascular treatment” OR “endovascular procedure*” OR “endovascular method*” OR “endovascular stenting” OR “endovascular adverse effect*” OR “endovascular thrombectomy” OR “endovascular embolization” OR “intra-arterial thrombolysis” OR “intraarterial thrombolysis” OR “intraarterial embolectomy” OR “intra-arterial thrombectomy” OR “intraarterial thrombectomy” OR “mechanical thrombolysis” OR “rheolytic thrombectomy” OR “Angiojet” OR “stent type retriever” OR “stent” OR “Trevo Retriever” OR “Penumbra device” OR “Solitaire revascularization Device” OR “balloon angioplasty”) AND (“Blood pressure decrease” OR “blood pressure reduction” OR “blood pressure lowering drugs” OR “antihypertensive*” OR “antihypertensive drug*” OR “beta blockers” OR “atenolol” OR “propanalol” OR “calcium channel blockers” OR “nimodipine” OR “nicardipine” OR “amilodipine” OR “felodipine” OR “isradipine” OR “nifedipine” OR “nisolodipine” OR “angiotensin-converting enzyme inhibitors” OR “ACE inhibitors” OR “captopril” OR “enalapril” OR “lisinopril” OR “perindopril” OR “Ramipril” OR “angiotensin receptor blocker*” OR “angiotensin receptor antagonist*” OR “candesartan” OR “losartan” OR “Telmisartan” OR “valsartan” OR “clonidine” OR “thiazide” OR “bendrofluazide” OR “Bendroflumethiazide” OR “hydrochrlothiazide” OR “HCT” OR “vasoactive” OR “nitrate” OR “glyceryl trinitrate” OR “GTN” OR “nitric Oxide Donors”) AND (“In patient*” OR “Inpatient*” OR “hospitalized patient*” OR “admitted patient*” OR “indoor patient*” OR “hospital patient*”)

PICO 5: In patients with acute ischaemic stroke not treated with reperfusion therapies (intravenous thrombolysis or mechanical thrombectomy) and with clinical deterioration, does induced hypertension by any vasopressor drug compared to no drug improve outcome?

(“ischaemic stroke” OR “ischemic stroke” OR “Intracranial Arteriosclerosis” OR “brain ischemia” OR “brain infarct*”) AND (“Low blood pressure” OR “hypotension”) AND (“blood pressure increase” OR “blood pressure rise” OR “vasoconstrictors” OR “dopamine” OR “dobutamine” OR “noradrenaline” OR “norepinephrine” OR “epinephrine” OR “adrenaline” OR “isoproterenol” OR “isoprenaline” OR “phenylephrine” OR vasopressor* OR hypertension)

PICO 6: In patients with acute ischaemic stroke, does continuing versus temporarily stopping previous oral blood pressure lowering therapy improve outcome?

PICO 7: In patients with acute intracerebral haemorrhage, does intensive blood pressure lowering with any vasodepressor drug compared to control improve outcome?

PICO 8: In patients with acute intracerebral haemorrhage, does continuing versus temporarily stopping previous oral antihypertensive therapy improve outcome?

("Acute stroke" OR "Acute cerebrovascular accident*" OR "CVA" OR "cerebrovascular apoplexy" OR "cerebrovascular infarct*" OR "cerebrovascular embolism" OR "cerebrovascular disorder" OR "brain ischemia" OR "brain infarct*" OR "brain haemorrhagic stroke" OR "ischaemic stroke" OR "cerebral embolism" OR "cerebral haemorrhage" OR "intracerebral haemorrhage" OR "ICH" OR "cardioembolic stroke" OR "hemiparesis" OR "hemiplegia" OR "Intracranial arteriosclerosis") AND ("Blood pressure decrease" OR "blood pressure reduction" OR "blood pressure lowering drugs" OR "antihypertensive*" OR "antihypertensive drug*" OR "beta blockers" OR "atenolol" OR "propanalol" OR "calcium channel blockers" OR "nimodipine" OR "nicardipine" OR "amilodipine" OR "felodipine" OR "isradipine" OR "nifedipine" OR "nisolodipine" OR "angiotensin-converting enzyme inhibitors" OR "ACE inhibitors" OR "captopril" OR "enalapril" OR "lisinopril" OR "perindopril" OR "Ramipril" OR "angiotensin receptor blocker*" OR "angiotensin receptor antagonist*" OR "candesartan" OR "losartan" OR "Telmisartan" OR "valsartan" OR "clonidine" OR "thiazide" OR "bendrofluazide" OR "Bendroflumethiazide" OR "hydrochrlothiazide" OR "HCT") AND ("Randomized Controlled Trials" OR "random allocation" OR "Controlled Clinical Trial*" OR "control groups" OR "clinical trials" OR "phase I" OR "phase II" OR "phase III" OR "phase IV" OR "double-blind method" OR "single-blind method" OR "randomized controlled trial" OR "RCT*" OR "random*" OR "quasi-random*" OR "pseudo-random*")

sFigure 1: Flow chart of studies included in the analyses and evidence profiles of all PICO questions

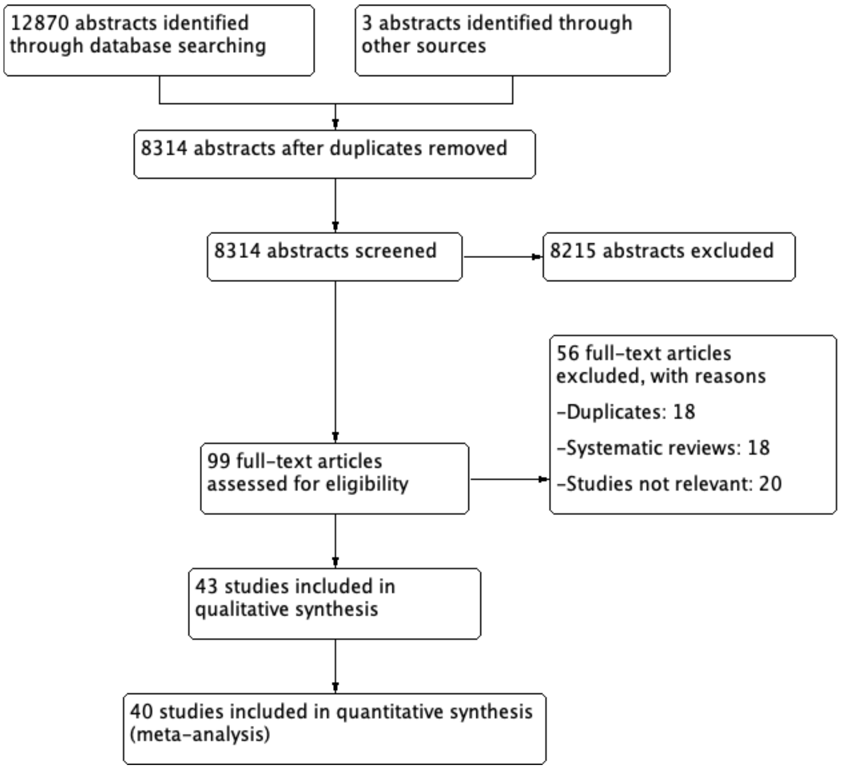

Supplement: sj-pdf-1-eso-10.1177_23969873211012133 - Supplemental material for European Stroke Organisation (ESO) guidelines on blood pressure management in acute ischaemic stroke and intracerebral haemorrhage [file sj-pdf-1-eso-10.1177_23969873211012133.pdf]
